# E3 ligase SlCOP1-1 stabilizes transcription factor SlOpaque2 and enhances fruit resistance to *Botrytis cinerea* in tomato

**DOI:** 10.1093/plphys/kiae404

**Published:** 2024-07-30

**Authors:** Guangtong Gao, Leilei Zhou, Jinying Liu, Peiwen Wang, Pichang Gong, Shiping Tian, Guozheng Qin, Weihao Wang, Yuying Wang

**Affiliations:** State Key Laboratory of Plant Diversity and Specialty Crops, Institute of Botany, Chinese Academy of Sciences, 100093 Beijing, China; China National Botanical Garden, 100093 Beijing, China; College of Advanced Agricultural Sciences, University of Chinese Academy of Sciences, 100049 Beijing, China; State Key Laboratory of Plant Diversity and Specialty Crops, Institute of Botany, Chinese Academy of Sciences, 100093 Beijing, China; China National Botanical Garden, 100093 Beijing, China; State Key Laboratory of Plant Diversity and Specialty Crops, Institute of Botany, Chinese Academy of Sciences, 100093 Beijing, China; China National Botanical Garden, 100093 Beijing, China; College of Advanced Agricultural Sciences, University of Chinese Academy of Sciences, 100049 Beijing, China; China National Botanical Garden, 100093 Beijing, China; College of Advanced Agricultural Sciences, University of Chinese Academy of Sciences, 100049 Beijing, China; State Key Laboratory of Plant Diversity and Specialty Crops, Institute of Botany, Chinese Academy of Sciences, 100093 Beijing, China; China National Botanical Garden, 100093 Beijing, China; College of Advanced Agricultural Sciences, University of Chinese Academy of Sciences, 100049 Beijing, China; China National Botanical Garden, 100093 Beijing, China; College of Advanced Agricultural Sciences, University of Chinese Academy of Sciences, 100049 Beijing, China; State Key Laboratory of Plant Diversity and Specialty Crops, Institute of Botany, Chinese Academy of Sciences, 100093 Beijing, China; China National Botanical Garden, 100093 Beijing, China; College of Advanced Agricultural Sciences, University of Chinese Academy of Sciences, 100049 Beijing, China; China National Botanical Garden, 100093 Beijing, China; College of Advanced Agricultural Sciences, University of Chinese Academy of Sciences, 100049 Beijing, China; State Key Laboratory of Plant Diversity and Specialty Crops, Institute of Botany, Chinese Academy of Sciences, 100093 Beijing, China; China National Botanical Garden, 100093 Beijing, China; College of Advanced Agricultural Sciences, University of Chinese Academy of Sciences, 100049 Beijing, China

## Abstract

CONSTITUTIVE PHOTOMORPHOGENIC 1 (COP1), a pivotal repressor in plant photomorphogenesis, has been extensively studied in various plant processes. However, the specific roles of COP1 in fruit remain poorly understood. Here, we functionally characterized SlCOP1-1 (also known as LeCOP1), an Arabidopsis (*Arabidopsis thaliana*) COP1 ortholog, in tomato (*Solanum lycopersicum*) fruit ripening and disease resistance. Despite the clear upregulation of *SlCOP1-1* during fruit ripening, knockout or overexpression (OE) of *SlCOP1-1* in tomatoes only minimally affected ripening. Intriguingly, these genetic manipulations substantially altered fruit resistance to the fungal pathogen *Botrytis cinerea*. Proteomic analysis revealed differential accumulation of proteins associated with fruit disease resistance upon *SlCOP1-1* knockout or OE. To unravel the mechanism of SlCOP1-1 in disease resistance, we conducted a screen for SlCOP1-1-interacting proteins and identified the stress-related bZIP transcription factor SlOpaque2. We provide evidence that SlOpaque2 functions in tomato resistance to *B. cinerea*, and SlCOP1-1-mediated mono-ubiquitination and stabilization of SlOpaque2 contributes to fruit resistance against *B. cinerea*. Our findings uncover a regulatory role of COP1 in controlling fruit disease resistance, enriching our understanding of the regulatory network orchestrating fruit responses to disease.

## Teaser

A repressor of plant photomorphogenesis stabilizes a stress-related transcription factor, an interaction that ultimately promotes fruit resistance to the fungal pathogen *Botrytis cinerea*.

## Introduction

Fleshy fruits, distinguished by their elevated nutritional value and delightful flavor, have gained substantial favor among consumers. However, these fruits are vulnerable to pathogen assaults both during cultivation and postharvest stages, resulting in reduced yields and economic losses (Prusky 1996; Cantu et al. 2008). In response to pathogen interactions, fruits employ diverse resistance pathways to resist pathogen invasion, and the orchestration of fruit defense responses is intricately regulated by various endogenous factors at multiple levels. These include transcriptional regulation (Tolosa and Zhang 2020; Yang et al. 2023), epigenome regulation (Xu et al. 2015; Zhou et al. 2023), and posttranscriptional regulation (Wang et al. 2017; Cheng et al. 2020). Understanding the mechanism underlying fruit disease resistance not only enriches the theory of plant disease resistance but also provides valuable insights for effective control of fruit disease.

Ubiquitination, an extensively studied posttranslational modification in plant defense response, plays a crucial regulatory role in disease resistance (Gough and Sadanandom 2021). This process involves a cascade of enzymes, including the ubiquitin-activating enzyme (E1), ubiquitin-conjugating enzyme (E2), and ubiquitin ligase [E3 (Scheffner et al. 1995)]. E3 ubiquitin ligase contributes to the specificity of substrate proteins, with numerous E3 ligases identified upstream of key components in disease resistance signaling (Duplan and Rivas 2014; Choi et al. 2022; Wang et al. 2023). The advantage of E3 ligase in dynamically and rapidly regulating the abundance of plant immune system components has prompted research in fruit to identify E3 ligases involved in disease response. However, to date, only a limited number of E3 ligases have been characterized in fruit disease resistance. Notable examples include the apple (*Malus domestica*) Plant U-box E3 ubiquitin ligase 29 (MdPUB29) and POZ/BTB CONTAINING-PROTEIN 1 (MdPOB1) in apple ring rot (Han et al. 2019a, b), the grapevine (*Vitis vinifera*) RING-H2 finger protein ATL E3 ubiquitin ligase 156 (VriATL156) in grape downy mildew (Vandelle et al. 2021), and the pear (*Pyrus bretschneideri*) ATL E3 ubiquitin ligase 18 (PbATL18) in pear anthracnose (Lin et al. 2023). Despite the recognized importance of E3 ligases in plant defense, there remains a substantial gap in our understanding of their roles in fruit disease resistance.

CONSTITUTIVE PHOTOMORPHOGENIC 1 (COP1) is one of the extensively studied RING-type E3 ligases in plants. It was initially identified as a repressor of photomorphogenesis in darkness by mediating the degradation of specific components in light signaling cascades (Deng et al. 1992; Osterlund et al. 2000; Seo et al. 2004). Subsequent investigations unveiled that COP1, in collaboration with various regulatory proteins, not only induces target protein degradation but also modulates target stability, participating in a diverse array of light-dependent or light-independent biological processes, such as stem elongation (Sharma et al. 2019), circadian rhythm (Yu et al. 2008), photoprotection (Tokutsu et al. 2019), gravitropism (Artz et al. 2019), hormone regulation (Shi et al. 2016; Blanco-Touriñán et al. 2020; Peng et al. 2022), and plant resistance (Gangappa and Kumar 2018; Lim et al. 2018). Although COP1 plays a pleiotropic role in plants, its function in horticultural fruits has not yet been systematically studied. Some reports indicate that COP1 or COP1-like proteins regulate color synthesis in tomatoes [*Solanum lycopersicum* (Liu et al. 2004)], apples (Li et al. 2012), pears (Wu et al. 2019), peaches [*Prunus persica* (Zhao et al. 2023)], and strawberries [*Fragaria × ananassa* (Liu et al. 2023)]. Additionally, heterologous expression of the eggplant (*Solanum melongena*) *SmCOP1* in tomato influenced ethylene signaling (Naeem et al. 2019). However, whether they are involved in other regulatory processes in fruits remains unclear.

Tomato fruit stands out as one of the most crucial horticultural crops. *Botrytis cinerea*, a notorious necrotrophic fungus, causes gray mold in numerous crops (Williamson et al. 2007; Zhang et al. 2021). The interaction between tomato fruits and *B. cinerea* serves as a model pathosystem for unraveling host cell defense mechanisms (Arie et al. 2007; Wang et al. 2017; Zhou et al. 2023). In this study, we identified 3 homologous *COP1* genes in tomatoes and explored the role of *SlCOP1-1*, the most similar to the Arabidopsis (*Arabidopsis thaliana*) *COP1* gene but hitherto uncharacterized in tomatoes. We generated stable CRISPR/Cas9-knockout mutants and *SlCOP1-1* overexpression (OE) lines, showing that SlCOP1-1 plays a more important role in fruit resistance to *B. cinerea* rather than in the regulation of fruit ripening. Compared to wild-type fruit, *Slcop1-1* mutants exhibited increased susceptibility to *B. cinerea*, while *SlCOP1-1* OE lines displayed enhanced disease resistance. Comparative proteomic analysis revealed that SlCOP1-1 regulates a set of proteins, including those involved in fruit disease resistance. Yeast 2-hybrid (Y2H) screening for SlCOP1-1-interacting proteins identified a bZIP transcription factor, SlOpaque2, as the direct target of SlCOP1-1. We provide evidence that SlOpaque2 is stabilized by SlCOP1-1 through mono-ubiquitination and functions downstream of SlCOP1-1 to govern disease resistance in tomato fruit.

## Results

### SlCOP1-1 exhibits increased expression during fruit ripening

**Figure 1.**
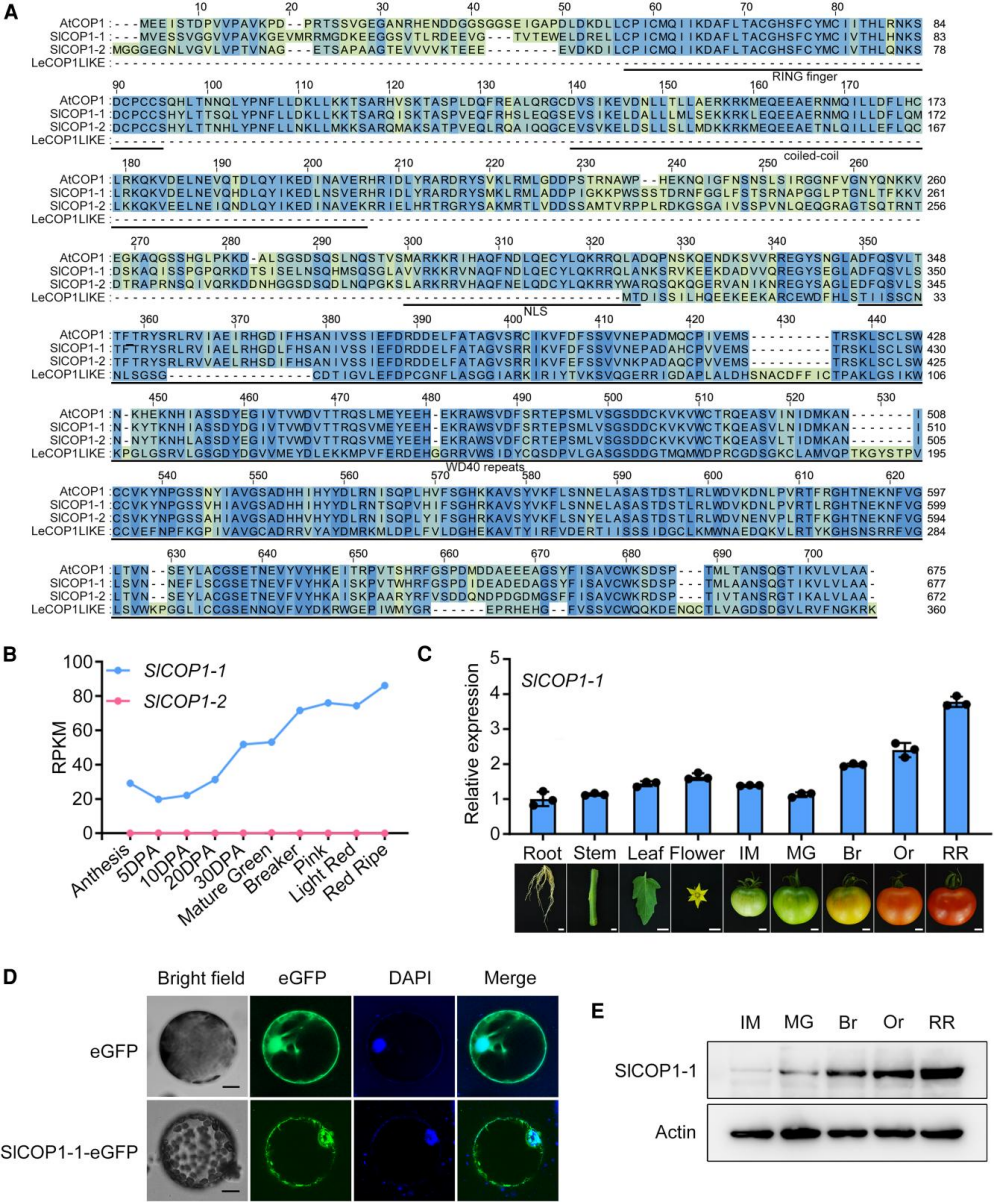
Identification and characterization of SlCOP1-1. **A)** Alignment between AtCOP1 and 3 putative tomato COP1 orthologs (SlCOP1-1, SlCOP1-2, LeCOP1LIKE). Functional motifs (RING finger, coiled-coil, WD40 repeats) and the nuclear localization signal (NLS) are underlined based on the AtCOP1structure. Blue indicates identical residues, yellow indicates differing residues, and shades between blue and yellow represent intermediate similarity. **B)** Expression profile of *SlCOP1-1* in tomato cv. Heinz. Data, based on 2 biological replicates from the Tomato Expression Atlas (TEA) database. RPKM, reads per kilobase per million mapped reads. DPA, days post anthesis. **C)** Expression of *SlCOP1-1* in the root, stem, leaf, flower, and fruit at various ripening stages in tomato cv. Ailsa Craig, as determined by RT-qPCR. Values represent means ± standard deviation (SD) of 3 independent experiments. *Actin* was used as an internal control. IM, immature; MG, mature green; Br, breaker; Or, orange; RR, red ripe. **D)** Subcellular localization of SlCOP1-1. Protoplasts from *Nicotiana benthamiana* leaves transiently expressing SlCOP1-1-eGFP were observed by confocal microscopy. 4′,6-diamidino-2-phenylindole (DAPI) was used for nuclear staining. Scale bars, 10 *μ*m. **E)** Western blot analysis of SlCOP1-1 in fruit at various ripening stages. Actin served as the protein loading control.

We used the BLAST tool embedded within the Sol Genomics Network (SGN; https://solgenomics.net/tools/blast/) to identify the tomato COP1 by querying the protein sequence of Arabidopsis COP1 (AtCOP1, AT2G32950). Three putative tomato COP1 orthologs (SlCOP1-1, also named LeCOP1, Solyc12g005950; SlCOP1-2, Solyc11g011980; LeCOP1LIKE, Solyc11g005190) were identified, sharing 76.5%, 72.31%, and 29.2% sequence identity with AtCOP1, respectively (Fig. 1A). Both SlCOP1-1 and SlCOP1-2 proteins have 3 conserved functional domains (RING finger, coiled-coil, WD40 repeats) and one nuclear localization signal (NLS), while LeCOP1LIKE only contains the WD40 repeats (Fig. 1A). Phylogenetic analysis confirmed that among the 3 COP1 orthologs, SlCOP1-1 is closely related to AtCOP1, followed by SlCOP1-2 and LeCOP1LIKE (Supplementary Fig. S1). A previous study has described the function of LeCOP1LIKE as a negative regulator of fruit pigmentation (Liu et al. 2004). Here, we focused on the function of SlCOP1-1 and SlCOP1-2 in tomato fruit.

First, we investigated the expressions of *SlCOP1-1* and *SlCOP1-2* in tomato fruit using the published transcriptome dataset, Tomato Expression Atlas (TEA; http://tea.solgenomics.net/) database, built on the tomato cv. Heinz (Shinozaki et al. 2018). As shown in Fig. 1B, *SlCOP1-1* displays an increased expression pattern during fruit development and ripening, while *SlCOP1-2* is barely detected in fruit (Fig. 1B), indicating that SlCOP1-2 may have limited or no role in the fruit-related process. We further conducted RT-qPCR to determine the expression of *SlCOP1-1* in various organs (root, stem, leaf, flower, and fruit at various ripening stages) in tomato cv. Ailsa Craig. It was confirmed that *SlCOP1-1* exhibits a continuously upregulated expression pattern during fruit ripening (Fig. 1C). These results suggest that *SlCOP1-1* might function in tomato fruit, and *SlCOP1-1* was chosen for further analysis in this study.

To observe the subcellular location of SlCOP1-1, we transiently expressed a fusion protein of SlCOP1-1 and enhanced Green Fluorescent Protein (eGFP) in *Nicotiana benthamiana* leaves. Confocal images showed that SlCOP1-1 is distributed in both cytoplasmic and nuclear compartments (Fig. 1D). This is consistent with the dual localization observed for the overexpressed AtCOP1 in transgenic *Arabidopsis*, which exhibits an elongated hypocotyl phenotype under light conditions (McNellis et al. 1994). Furthermore, we examined the protein level of SlCOP1-1 during fruit ripening using a western blot by a SlCOP1-1-specific antibody. Consistent with its transcription level, SlCOP1-1 protein gradually increased with fruit ripening (Fig. 1E), confirming its potential role in fruit ripening.

### SlCOP1-1 positively regulates tomato fruit disease resistance to *B. cinerea*

**Figure 2.**
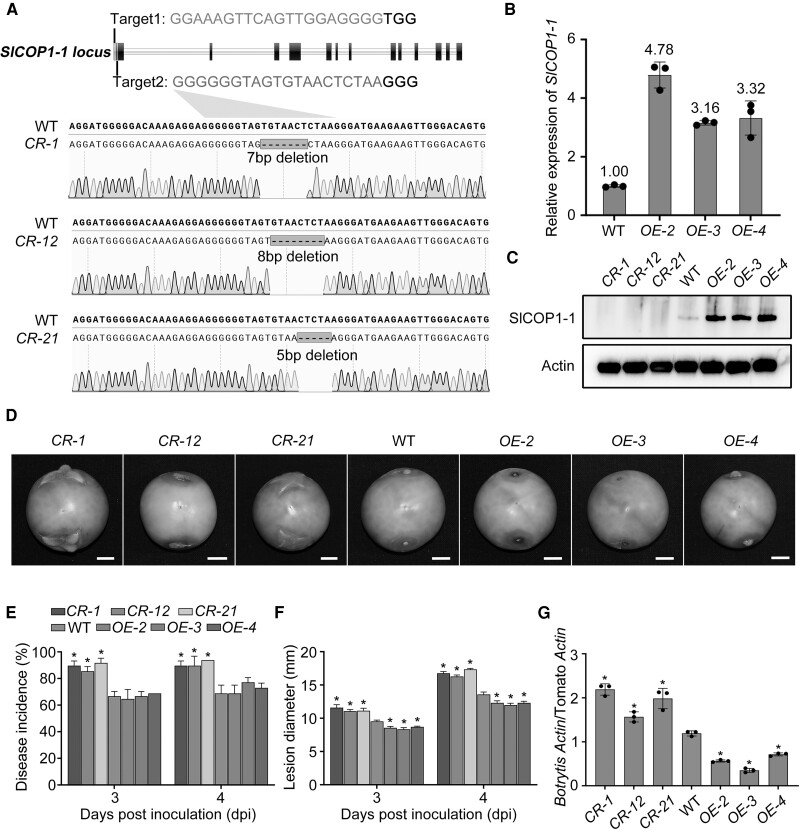
SlCOP1-1 positively regulates fruit resistance against *B. cinerea* in tomato. **A)** Genotyping of mutations induced by the CRISPR/Cas9 genome-editing system in *Slcop1-1* mutants. Two specific targets on the first exon were designed, with target 1 and 2 indicated by red and blue letters, respectively. The black letter following the target sequences denotes the protospacer adjacent motif (PAM). Sequences of the genomic region flanking the 2 targets in the wild-type (WT) and *Slcop1-1* mutants (*CR-1*, *CR-12*, and *CR-21*) are shown. **B)** Changes in *SlCOP1-1* mRNA abundance in *SlCOP1-1* overexpression (OE) fruits (*OE-2*, *OE-3*, and *OE-4*), as determined by RT-qPCR. *Actin* was used as an internal control. Values are means ± standard deviation (SD) of 3 biological replicates. **C)** Changes in SlCOP1-1 protein levels in *Slcop1-1* mutants and *SlCOP1-1* OE fruits, as determined by immunoblotting analysis using SlCOP1-1 specific antibody. **D)** Representative photograph of the detached *Slcop1-1* mutants, WT, and *SlCOP1-1* OE fruits inoculated with *B*. *cinerea* for 3 d. Scale bars, 1 cm. **E)** to **G)** Changes in disease incidences E), lesion diameters F), and fungal biomasses G) of *Slcop1-1* mutants and *SlCOP1-1* OE fruits inoculated with *B*. *cinerea* for 3 d. In G), the relative amount of *B*. *cinerea* was evaluated by the ratio of *B*. *cinerea Actin* to tomato *Actin* gene level as determined by qPCR. In E), F), and G), values are means ± SD of 3 biological replicates, with each containing at least 10 fruits. Asterisks denote statistically significant differences (*, *P* < 0.05, Student's *t*-test).

To further understand the biological function of SlCOP1-1, we generated *Slcop1-1* mutants in tomatoes using CRISPR/Cas9 genome-editing technology. Two specific targets were designed to introduce mutations into the *SlCOP1-1* locus. Three distinct homozygous *Slcop1-1* mutant lines (*CR-1*, *CR-12*, and *CR-21*) were obtained, with 7-, 8-, and 5-bp deletions in the second target, resulting in nonfunctional truncated SlCOP1-1 proteins (Fig. 2A). No off-target editing was detected in these mutants (Supplementary Fig. S2). Simultaneously, *SlCOP1-1* OE lines were developed using the cauliflower mosaic virus (CaMV) 35S promoter, and *OE-2*, *OE-3*, and *OE-4* were selected for analysis.

Expression analysis of SlCOP1-1 in both *Slcop1-1* mutant and *SlCOP1-1* OE lines showed that the expression of SlCOP1-1 in *SlCOP1-1* OE lines exhibits at least a 3-fold increase both in mRNA and protein levels compared to the wild type. In contrast, SlCOP1-1 protein was undetectable in *Slcop1-1* mutants (Fig. 2B and C; Supplementary Fig. S3). Moreover, the expressions of other tomato *COP1* orthologs (*SlCOP1-2* and *LeCOP1LIKE*) were unaffected (Supplementary Fig. S4). These results confirm the successful construction of the *Slcop1-1* mutants and *SlCOP1-1* OE lines in tomatoes.

Next, we observed the ripening phenotypes of *Slcop1-1* mutants and *SlCOP1-1* OE lines during the cause of fruit ripening. As shown in Supplementary Fig. S5, a slight but significant difference (*P* < 0.05, Student's *t-*test) was observed in the days of fruit reaching the breaker stage between *Slcop1-1* mutants or *SlCOP1-1* OE lines and the wild type. *Slcop1-1* mutants exhibited delayed ripening, while *Slcop1-1* OE lines showed accelerated ripening. To further dig out the molecular events underlying fruit ripening, we examined the expression of several well-known ripening-related genes, including *ACC synthase 2* (*ACS2*), *ACC synthase 4* (*ACS4*), *phytoene synthase* (*PSY*), as well as *phytoene desaturase* (*PDS*). However, no significant changes (*P* < 0.05, Student's *t-*test) were observed in the expression of these genes in fruit at the breaker stage from either *Slcop1-1* mutants or *SlCOP1-1* OE lines compared to the wild type (Supplementary Fig. S5). These data suggest that SlCOP1-1 may play a minor role in regulating fruit ripening and the effects of SlCOP1-1 on fruit ripening might occur at the posttranscriptional level.

We further investigated whether SlCOP1-1 participates in fruit resistance to *Botrytis cinerea* by inoculating fruits harvested at 33 d post-anthesis (dpa). The disease incidence and lesion diameter were measured at 3 d post inoculation (dpi) when the obvious disease symptoms occurred in almost all fruits. Strikingly, *Slcop1-1* mutants displayed higher disease incidence and larger lesions compared to the wild-type and *SlCOP1-1* OE lines. Meanwhile, *SlCOP1-1* OE lines exhibited significantly smaller lesions (*P* < 0.05, Student's *t-*test) than the wild type (Fig. 2D, E and F). We concurrently evaluated the fungal biomass in fruit based on the ratio of *B*. *cinerea Actin* to tomato *Actin* using qPCR amplification. Consistent with the disease symptoms, *B*. *cinerea* biomass was increased in *Slcop1-1* mutant fruits and decreased in *SlCOP1-1* OE fruit compared to the wild type (Fig. 2G). These results demonstrate that SlCOP1-1 plays a crucial role in positively regulating fruit resistance to *B*. *cinerea*.

### SlCOP1-1 modulates the accumulation of proteins associated with fruit disease resistance

**Figure 3.**
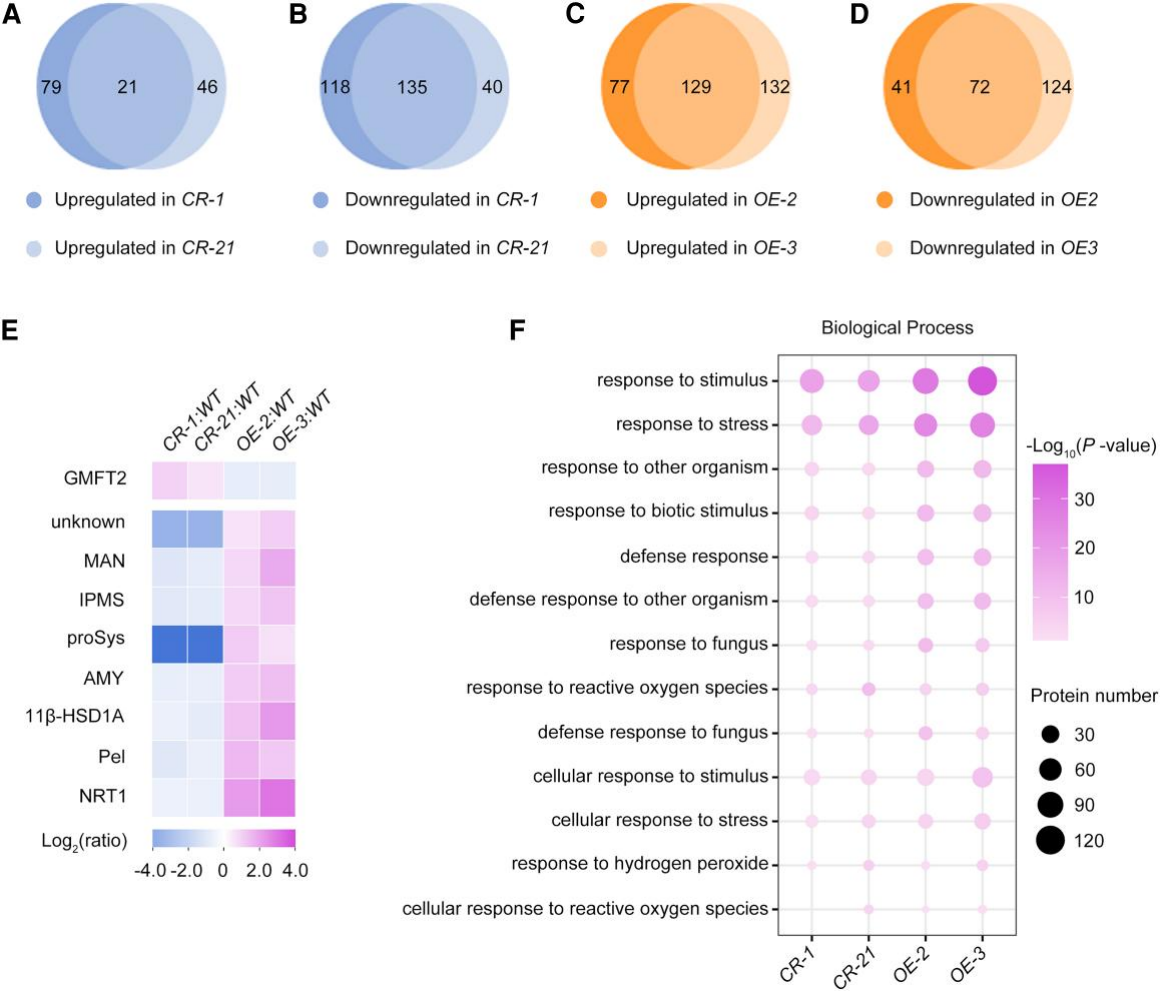
Quantitative proteome reveals altered levels of proteins associated with fruit disease resistance in *Slcop1-1* mutant and *SlCOP1-1* overexpression (OE) fruits compared to the wild type (WT). **A)** to **D)** Venn diagrams depicting the overlaps of upregulated proteins (A, C) and downregulated proteins (B, D), between 2 *Slcop1-1* mutants, *CR-1* (left) and *CR-21* (right), or 2 *SlCOP1-1* OE lines, *OE-2* (left), *OE-3* (right), compared to WT, respectively. **E)**, Heatmap showing the expression levels of nine proteins with opposing expression changes in *Slcop1-1* mutants and *SlCOP1-1* OE lines. GMFT2, Glucomannan 4-beta-mannosyltransferase 2; MAN, mannan endo-1,4-beta-mannosidase; IPMS, Isopropylmalate synthase; proSys, prosystemin; AMY, 1,4-alpha-glucan-maltohydrolase; 11β-HSD1A, 11-beta-hydroxysteroid dehydrogenase 1A; Pel, pectate lyase; NRT1, NRT1/PTR FAMILY 1.1. **F)** Heatmap displaying enriched Gene Ontology (GO) terms associated with the resistant response to disease in *Slcop1-1* mutant (*CR-1*, *CR-21*) and *SlCOP1-1* OE (*OE-2*, *OE-3*) fruits (*P* < 0.05, Fisher's exact test). Proteins isolated from 2 *Slcop1-1* mutants, 2 *SlCOP1-1* OE lines, and the wild-type fruits at 33 d post anthesis (dpa) were labeled with Tandem Mass Tag (TMT) and subjected to nanoLC-MS/MS. Differentially expressed proteins were identified by 3 independent proteome analyses, with each protein showing a fold change >1.5 or < 0.67 (*P* < 0.05, background-based *t*-test).

To decipher the molecular mechanism underlying SlCOP1-1-mediated resistance to *B. cinerea* in tomato fruit, we performed a comparative proteome analysis of the wild-type, *Slcop1-1* mutants, and *SlCOP1-1* OE fruits. Proteins from fruits harvested at 33 dpa from *CR-1*, *CR-21*, *OE-2*, and *OE-3*, as well as those from the wild type, were labeled with Tandem Mass Tag (TMT) reagent and submitted to NanoLC-MS/MS analysis. In *CR-1*, 353 proteins were identified as differentially expressed, including 100 upregulated and 253 downregulated proteins. Similarly, in *CR-21*, 242 proteins showed differential expression, with 67 upregulated and 175 downregulated proteins. The overlap between the 2 *Slcop1-1* mutants revealed 21 upregulated and 135 downregulated proteins [fold change >1.5 or <0.67, *P* < 0.05 (Fig. 3A and B; Supplementary Data Set 1]. In *OE-2*, 319 proteins were differentially expressed, comprising 206 upregulated and 113 downregulated proteins. In *OE-3*, 457 proteins were differentially expressed, with 261 upregulated and 196 downregulated proteins. Notably, 129 upregulated and 72 downregulated proteins overlapped between the 2 *SlCOP1-1* OE lines [fold change >1.5 or <0.67, *P* < 0.05 (Fig. 3C and D; Supplementary Data Set 1].

Next, 21 upregulated and 135 downregulated proteins in *Slcop1-1* mutants were overlapped with the 72 downregulated and 129 upregulated proteins in *SlCOP1-1* OE lines, respectively. Nine proteins with opposite expression patterns in *Slcop1-1* mutants and *SlCOP1-1* OE lines were identified (Fig. 3E), indicating their likely direct regulation by SlCOP1-1. These proteins included cell wall modification enzymes such as mannan endo-1,4-beta-mannosidase (MAN), pectate lyase (Pel), and Glucomannan 4-beta-mannosyltransferase 2 (GMFT2), nutrient transporters and metabolic enzymes like protein NRT1/PTR FAMILY 1.1 (NRT1), isopropylmalate synthase (IPMS), and 1,4-alpha-glucan-maltohydrolase (AMY), as well as other resistant proteins such as prosystemin [proSys (Fig. 3E; Supplementary Data Set 1)].

Furthermore, we performed a Gene Ontology (GO) enrichment analysis of differentially expressed proteins to identify the biological processes regulated by SlCOP1-1. This analysis highlighted processes such as “response to high/low intensity light,” “starch metabolic process,” “regulation of chlorophyl biosynthetic process” (Supplementary Data Set 2), suggesting a broader role for SlCOP1 beyond light-related functions. Furthermore, we focused on the enriched terms associated with disease resistance, such as “response to stimulus,” “response to stress” and “response to other organism.” It is shown that these terms were enriched in *SlCOP1-1* OE lines and *Slcop1-1* mutants (Fig. 3F; Supplementary Data Set 2). This result suggests that SlCOP1-1 directly or indirectly regulates these proteins to modulate tomato fruit disease resistance.

### SlCOP1-1 directly interacts with SlOpaque2 in nuclei

**Figure 4.**
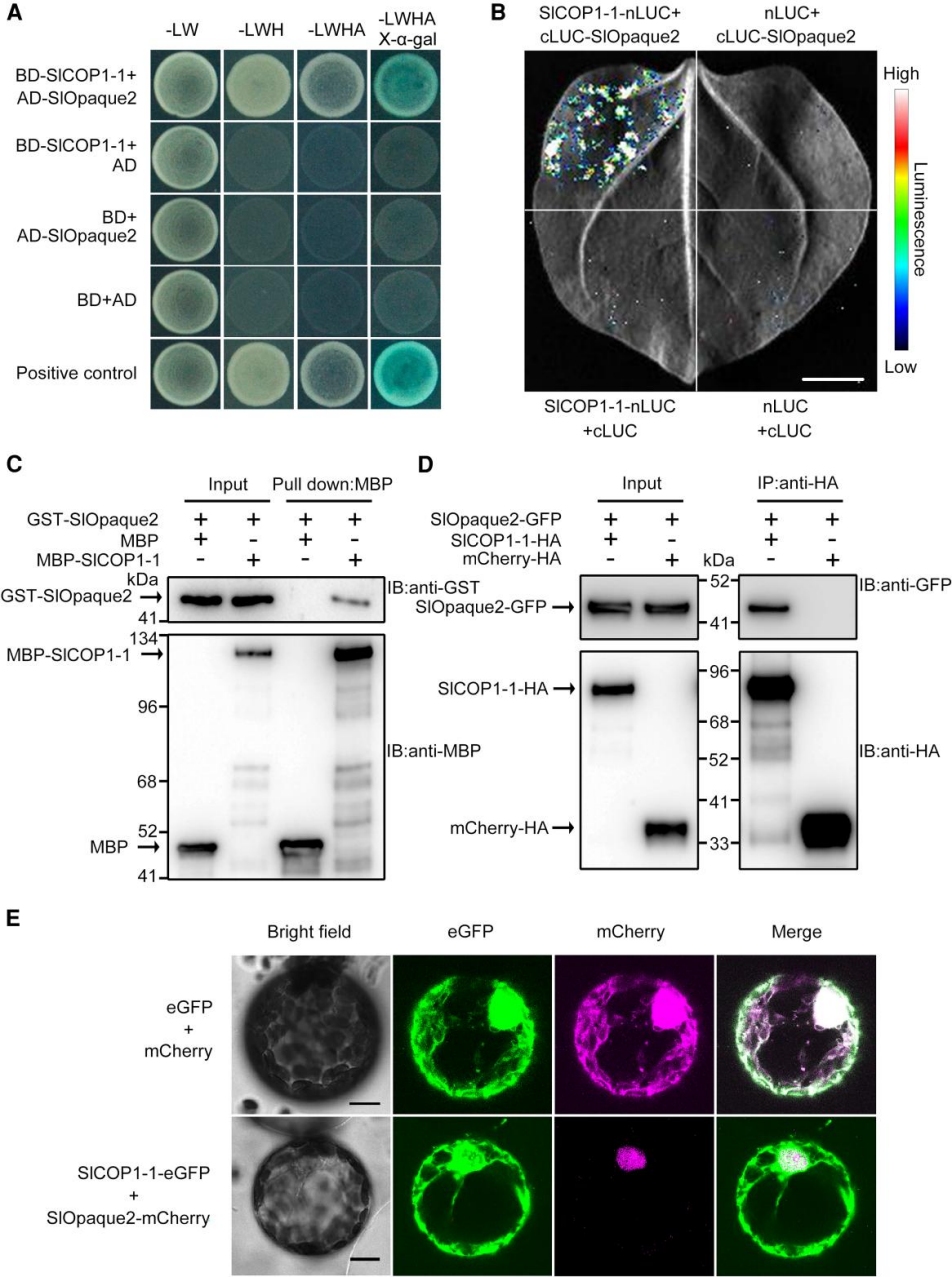
SlCOP1-1 interacts with SlOpaque2 in the nucleus. **A)** Yeast 2-hybrid assay confirming the interaction between SlCOP1-1 and SlOpaque2. SlCOP1-1 fused with the BD domain of GAL4 (BD-SlCOP1-1) was co-expressed with SlOpaque2 fused with the AD domain of GAL4 (AD-SlOpaque2) in yeast. The recombinant yeasts were selected on SD/-Leu/-Trp (-LW), SD/-Leu/-Trp/-Trp (-LWH), and SD/-Leu/-Trp/-His/-Ade (-LWHA) cultural media, with or without X-α-gal. Negative controls include parallel co-expression of BD-SlCOP1-1/AD, BD/AD-SlOpaque2, and AD/BD. **B)** Luciferase complementation imaging assay revealing the interaction between SlCOP1-1 and SlOpaque2. SlCOP1-1 fused with the N-terminus of luciferase (SlCOP1-1-nLUC) was transiently co-expressed SlOpaque2 fused with the C-terminus of luciferase (cLUC-SlOpaque2) in *Nicotiana benthamiana* leaves. Scale bar, 1 cm. **C)** A pull-down assay revealing the interactions between SlCOP1-1 and SlOpaque2. Recombinant GST-SlOpaque2, MBP-SlCOP1-1, and MBP tag protein (as a negative control) were mixed as indicated, and incubated with anti-MBP magnetic beads. Immunoblots were conducted to detect the eluted proteins using anti-MBP or anti-GST antibodies. IB, immunoblot. **D)** Co-immunoprecipitation assay revealing the interaction between SlCOP1-1 and SlOpaque2. SlCOP1-1-HA was transiently co-expressed with SlOpaque2-GFP in *N. benthamiana* leaves. The mCherry-HA served as a negative control. Total proteins extracted from transformed leaves were immunoprecipitated with anti-HA beads, followed by immunoblot analysis using anti-GFP or anti-HA antibodies. IP, immunoprecipitation. **E)** Subcellular colocalization of SlCOP1-1 and SlOpaque2. Fusion proteins of SlCOP1-1-eGFP and SlOpaque2-mCherry were co-expressed in *N. benthamiana* leaves. Non-fused eGFP and mCherry were used as a control. Scale bars, 10 *μ*m.

As COP1 is an E3 ligase, we hypothesized that the downstream substrates of SlCOP1-1 might play a role in regulating disease resistance in tomato fruit. Thus, we conducted a Y2H screen to identify the potential partner that interacts with SlCOP1-1. In total, 104 colonies were isolated on the selective medium, resulting in the identification of 65 proteins (Supplementary Table S1). Further Y2H validation using their full-length coding sequence (CDS) revealed 5 candidate SlCOP1-1-interacting proteins, including the bZIP transcription factor SlOpaque2 [Solyc08g022080 (Supplementary Fig. S6; Fig. 4A)]. Given the previous study in maize (*Zea mays*) that demonstrated the involvement of Opaque2 in plant disease resistance (Loesch et al. 1976), we chose the SlOpaque2 protein for analysis.

To validate the interaction between SlCOP1-1 and SlOpaque2, we performed a split luciferase complementation imaging (LCI) assay. SlCOP1-1 and SlOpaque2, fused with the N- and C-termini of luciferase, respectively, were transiently co-expressed in *N. benthamiana* leaves. An obvious luminescence was observed in the leaves co-expressing SlCOP1-1-nLuc and cLuc-SlOpaque2, whereas no signals were detected in the negative controls (Fig. 4B). We subsequently carried out a pull-down assay using prokaryotically expressed MBP-SlCOP1-1 and GST-SlOpaque2 recombinant proteins. As shown in Fig. 4C, GST-SlOpaque2, but not the MBP tag protein, were observed to bind to MBP-SlCOP1-1, indicating the in vitro interaction between SlCOP1-1 and SlOpaque2. Moreover, we performed a co-immunoprecipitation (Co-IP) assay using proteins extracted from *N. benthamiana* leaves co-expressing SlCOP1-1-HA and SlOpaque2-GFP. Indeed, SlOpaque2-GFP was successfully co-immunoprecipitated with SlCOP1-1-HA by anti-HA beads (Fig. 4D), confirming the in vivo interaction between SlCOP1-1 and SlOpaque2.

We finally conducted a colocalization analysis of SlOpaque2 with SlCOP1-1 in *N. benthamiana* leaves. SlOpaque2 fused with red fluorescent protein mCherry and SlCOP1-1 fused with eGFP were transiently co-expressed in *N. benthamiana* leaves. As shown in Fig. 4E, SlOpaque2-mCherry showed a nucleus-localization signal, while SlCOP1-1-eGFP signals were observed in both cytoplasm and nucleus. The red signal from SlOpaque2-mCherry co-localized with the green signal from SlCOP1-1-eGFP in the nucleus, demonstrating the subcellular colocalization of SlCOP1-1 and SlOpaque2. Collectively, these data suggest that SlCOP1-1 can directly interact with SlOpaque2 in nuclei.

### SlCOP1-1 mono-ubiquitinates and stabilizes the SlOpaque2 protein

**Figure 5.**
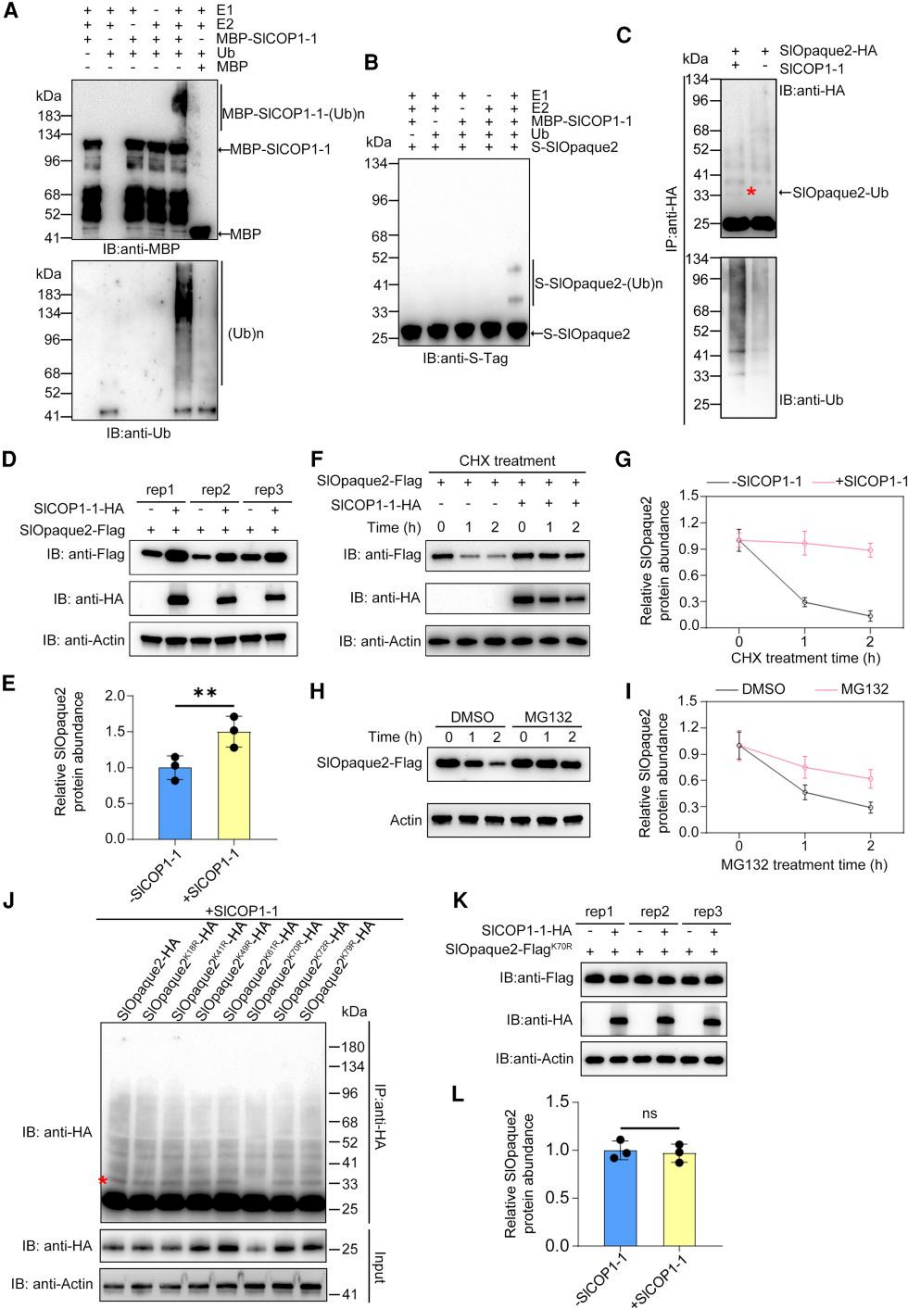
SlCOP1-1 oligo-ubiquitinates and stabilizes SlOpaque2. **A)**, **B)***In vitro* ubiquitination assay demonstrating SlCOP1-1 as a ubiquitin ligase A) and the ubiquitination of SlOpaque2 by SlCOP1-1 B). Ubiquitination reactions were conducted in the presence (+) or absence (−) of His-tagged ubiquitin (Ub), E1, E2, MBP-tagged SlCOP1-1 (MBP-SlCOP1-1), or S-tagged SlOpaque2 (S-SlOpaque2). The reaction products were subjected to immunoblot analysis using anti-MBP, anti-S-Tag, or anti-Ub antibodies. MBP protein was used as the negative control. (Ub)n, polyubiquitin chain. **C)** In vivo ubiquitination of SlOpaque2 by SlCOP1-1. SlOpaque2-HA were co-expressed with SlCOP1-1 in *Nicotiana benthamiana* leaves. Total proteins extracted from transformed leaves were immunoprecipitated with anti-HA beads, followed by immunoblot analysis using anti-HA or anti-Ub antibodies. IB, immunoblot. IP, immunoprecipitation. Red asterisk indicates the mono-ubiquitinated band. **D)**, E) Effect of SlCOP1-1 on the protein stability of SlOpaque2. The SlOpaque2-Flag was expressed in the presence (+) or absence (−) of SlCOP1-1-HA in *N. benthamiana* leaves. **F)**, **G)** Degradation rate analysis of SlOpaque2 in the presence (+) or absence (−) of SlCOP1-1. Co-expression of SlOpaque2-Flag with or without SlCOP1-1-HA was performed in *N. benthamiana* leaves, followed by treatment with translation inhibitor cycloheximide (CHX). **H)**, **I)** Stability analysis of SlOpaque2. SlOpaque2-Flag was expressed in *N. benthamiana* leaves followed by treatment with or without the proteasome inhibitor MG132. DMSO, the solvent for MG132, served as a control. For D), F), and H), total protein extracted from the transformed leaves was subjected to immunoblotting analysis using anti-HA or anti-Flag antibodies. Actin was used as the loading control. For E), G), and I), quantification of the immunoblot bands were performed by Image J software. Values are means ± standard deviation (SD) of 3 independent experiments. **J)** Screening for SlOpaque2 site ubiquitinated by SlCOP1-1 via in vivo ubiquitination assay. All seven lysine (K) sites were individually mutated to arginine (R). The HA-tagged SlOpaque2 variant forms were co-expressed with SlCOP1-1 in *N. benthamiana* leaves and subjected to ubiquitination analysis as described in C). Red asterisk indicates the mono-ubiquitinated bands. **K)**, **L)** Protein stability analysis of variant SlOpaque2^K70R^. The variant SlOpaque2^K70R^-Flag were co-expressed with SlCOP1-1-HA in *N. benthamiana* leaves followed by immunoblot analysis K) and quantification L) as described in D) and E). For E) and L), asterisks indicate statistically significant differences (*, *P* < 0.05, **, *P* < 0.01, Student's *t*-test). ns, not significant.

To test our hypothesis that SlOpaque2 functions as a substrate for SlCOP1-1, we carried out an in vitro ubiquitination assay to determine whether SlCOP1-1 ubiquitinates SlOpaque2. We first detected the E3 ligase activity of SlCOP1-1 by incubating the MBP-tagged recombinant SlCOP1-1 protein (MBP-SlCOP1-1) with wheat (*Triticum aestivum*) E1, human E2, and *Arabidopsis* ubiquitin (Ub) in vitro. Immunoblot analysis, employing anti-MBP or anti-Ub antibodies, revealed high molecular mass bands specifically observed in the intact reaction set but absent in the negative controls (Fig. 5A), indicating the presence of ubiquitinated MBP-SlCOP1-1 proteins. These results confirmed the possession of E3 ligase activity by SlCOP1-1 and its capacity for self-ubiquitination in vitro. We then detected the ubiquitination of SlOpaque2 catalyzed by SlCOP1-1 in vitro. S-tagged recombinant SlOpaque2 (S-SlOpaque2) was subjected to the in vitro ubiquitination assay, followed by immunoblotting analysis using anti-S-Tag antibody. Notably, 2 ubiquitinated bands were observed, with a molecular weight increase of approximately 12 kDa [roughly equal to the molecular weight of a single His-Ub (Fig. 5B)], indicating that SlCOP1-1 can oligo-ubiquitinate SlOpaque2 in vitro.

To validate that SlCOP1-1 indeed mediates the oligo-ubiquitination of SlOpaque2, we further detected the ubiquitination of SlOpaque2 in vivo by transiently co-expressing SlCOP1-1 and SlOpaque2-HA in *N. benthamiana* leaves. After immunoprecipitation of total leaf extracts with anti-HA, immunoblotting analysis using anti-HA or anti-Ub antibodies revealed a prominent ubiquitinated band, corresponding to the molecular weight of SlOpaque2 plus one Ub, in samples co-expressing SlCOP1-1 and SlOpaque2-HA (Fig. 5C). This indicated the presence of “mono-ubiquitinated” SlOpaque2-HA catalyzed by SlCOP1-1. The SlCOP1-1-mediated mono-ubiquitinated band of SlOpaque2 was also observed in *SlCOP1-1* OE lines but not in *Slcop1-1* mutants, confirming the role of SlCOP1-1 in mono-ubiquitinating SlOpaque2 in tomatoes (Supplementary Fig. S7). Although we could not accurately distinguish more oligo-ubiquitinated bands, as observed in the in vitro assay, from numerous polyubiquitinated bands of SlOpaque2 in vivo, these results convincingly demonstrated the capacity of SlCOP1-1 to mono-ubiquitinate SlOpaque2 in vivo.

Since E3 ubiquitin ligase-mediated protein mono-ubiquitination is often associated with protein stability (Scheffner et al. 1995), we investigate the impact of SlCOP1-1 on the protein level of SlOpaque2 by transiently co-expressing SlCOP1-1-HA and SlOpaque2-Flag in *N. benthamiana* leaves. As expected, the results showed that co-expression of SlCOP1-1 and SlOpaque2 did not induce a reduction in SlOpaque2 protein; instead, it rendered SlOpaque2 protein more stable, exhibiting a roughly 50% increase in protein abundance compared to the expression of SlOpaque2 alone (Fig. 5D and E). We then monitored the degradation rate of SlOpaque2 by introducing the translation inhibitor cycloheximide (CHX) to the co-expressing system. As shown in Fig. 5F and G, SlOpaque2 underwent rapid degradation in *N. benthamiana* following CHX treatment. By contrast, the co-expression of SlOpaque2 with SlCOP1-1 substantially attenuated SlOpaque2 degradation (67% to 73%) compared to the expression of SlOpaque2 alone. Further analysis showed that the degradation of SlOpaque2 could be decreased (29% to 33%) upon the application of the proteasome inhibitor MG132 (Fig. 5H and I). These results demonstrated that SlOpaque2 degrades via the ubiquitin-proteasome system and SlCOP1-1 plays a pivotal role in stabilizing SlOpaque2.

To identify the key ubiquitination site contributing to SlOpaque2 ubiquitination and stability mediated by SlCOP1-1, we conducted a site-directed mutagenesis analysis for all seven lysine residues (substitution of lysine [K] by arginine [R]) in SlOpaque2 protein. Co-expressing SlCOP1-1 with the mutant SlOpaque2 in *N. benthamiana* leaves revealed that, compared with individual mutations at K18, K41, K49, K61, K70, K72, and K79, the mutation at K70 specifically led to the loss of “mono-ubiquitinated” band and a substantial reduction in SlOpaque2 protein levels (Fig. 5J), indicating the critical role of K70 in SlCOP1-1-mediated SlOpaque2 ubiquitination and stability. Furthermore, we assessed the influence of SlCOP1-1 presence on the SlOpaque2^K70R^ variant protein stability. Indeed, the presence of the SlCOP1-1 protein did not alter the levels of the SlOpaque2^K70R^ variant protein (Fig. 5K and L). Taken together, these data indicated that K70 serves as the key site responsible for SlCOP1-1-mediated stability of SlOpaque2.

### SlOpaque2 enhances tomato resistance to *B. cinerea*

**Figure 6.**
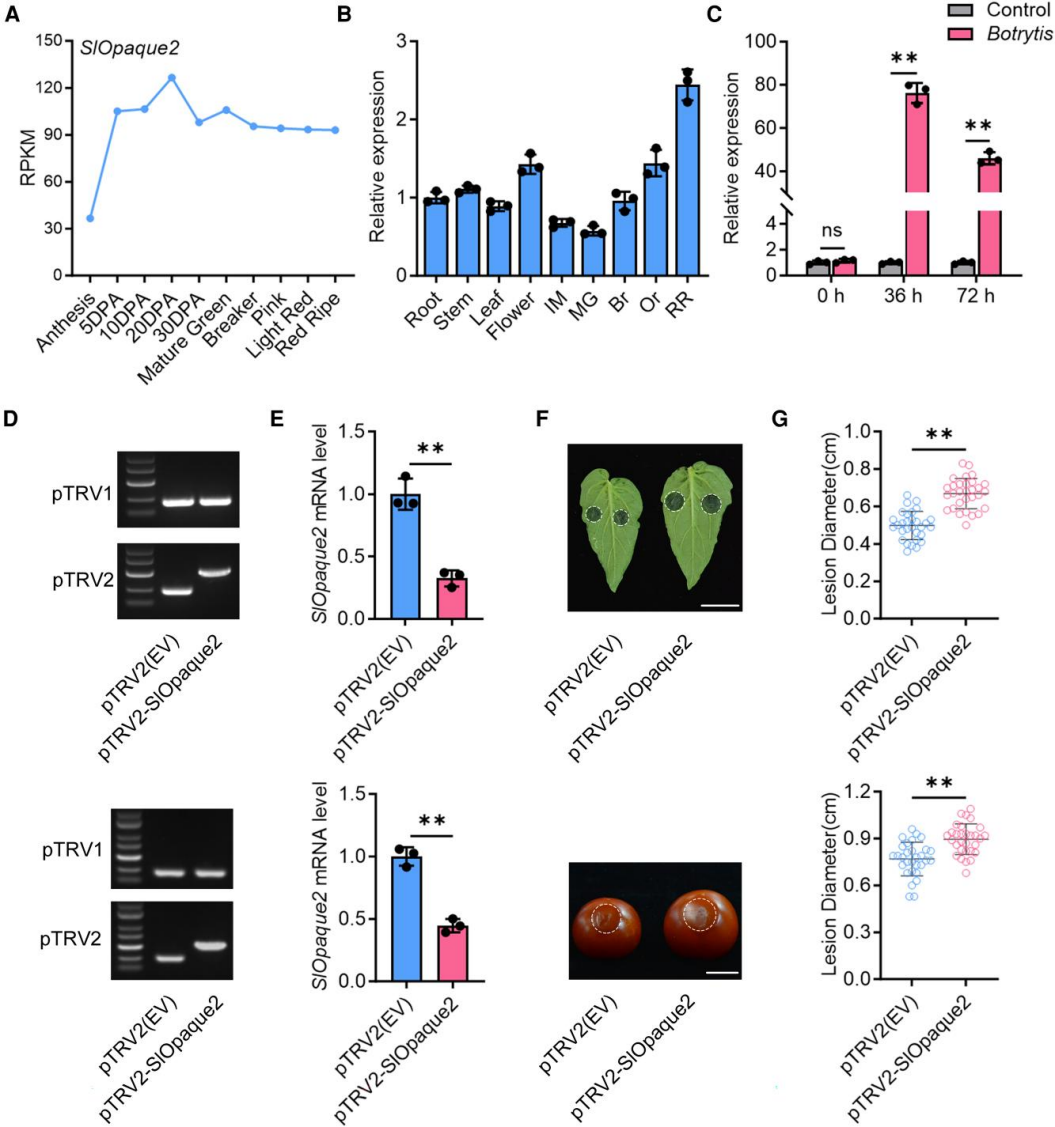
SlOpaque2 is involved in *Botrytis cinerea* disease resistance. **A)** Expression profiles of *SlOpaque2* in tomato cv. Heinz, based on 2 biological replicates from the Tomato Expression Atlas (TEA) database. RPKM, reads per kilobase per million mapped reads. DPA, days post anthesis. **B)***SlOpaque2* expression in root, stem, leaf, flower, and fruit at various ripening stages in tomato cv. Ailsa Craig. IM, immature; MG, mature green; Br, breaker; Or, orange; RR, red ripe. **C)***SlOpaque2* expression in fruits with or without *B. cinerea* inoculation at indicated times. h, hours. **D)** PCR amplification confirming the presence of virus vectors pTRV1, pTRV2, and pTRV2-*SlOpaque2* in tomato leaves (upper) and fruits (lower) post-Virus-Induced Gene Silencing (VIGS). **E)***SlOpaque2* expression in tomato leaves (upper) and fruits (lower) after VIGS. **F)**, **G)** Disease symptom F) and lesion diameters G) in *SlOpaque2*-silenced leaves (upper) and fruits (lower) inoculated with *B. cinerea* for 3 d. In B), C), and E), gene expression was determined using RT-qPCR. *Actin* served as an internal control. Values represent means ± standard deviation (SD) from 3 independent experiments. In G), values represent means ± SD (*n* = 30 inoculation sites in 15 leaves or fruits). In C), E) and G), asterisks indicate statistically significant differences (*, *P* < 0.05, **, *P* < 0.01, Student's *t-*test). ns, not significant.

Previous studies have demonstrated that Opaque2 plays a positive regulatory role in kennel nutrition accumulation and plant disease resistance in maize (Loesch et al. 1976). However, the functions of Opaque2 in other plants, including tomato, remain unexplored. Expression data from the TEA database (Shinozaki et al. 2018) revealed a relatively high expression of the *SlOpaque2* gene during tomato fruit development and ripening (Fig. 6A). RT-qPCR analysis further confirmed that *SlOpaque2* exhibited stable and elevated gene expression in both vegetable and fruit organs (Fig. 6B), suggesting its potential roles in fruit-related processes. Notably, *SlOpaque2* expression was significantly increased (*P* < 0.05, Student's *t*-test) in tomato fruits following inoculation with *B. cinerea* (Fig. 6C), indicating its involvement in modulating disease resistance in tomato against this pathogen.

To further investigate the role of *SlOpaque2* in the regulation of tomato resistance to *B*. *cinerea*, we employed virus-induced gene silencing (VIGS) to knock-down the *SlOpaque2* gene in tomato and assessed its impact on plant defense against *B. cinerea*. An optimized cDNA fragment of *SlOpaque2* was cloned into the pTRV2 vector and co-injected with the pTRV1 vector into the tomato cv. Micro-Tom plant. As shown in Fig. 6D, RNA fragments transcribed from the virus vectors pTRV1, pTRV2, and pTRV2-SlOpaque2 could be detected in *SlOpaque2*-silenced plants. Meanwhile, the transcript level of *SlOpaque2* in *SlOpaque2*-silenced plants was reduced by approximately 68% in leaves and 55% in fruits compared to the control (Fig. 6E), confirming that *SlOpaque2* gene was successfully silenced in tomato. Fruit phenotype and gene expression analysis on ripening-related genes (i.e. *ACS2*, *ACS4*, *PSY1*, and *PDS*) showed no obvious changes in fruit ripening after *SlOpaque2* knock-down (Supplementary Fig. S8). We then carried out a *B. cinerea* inoculation assay on *SlOpaque2*-silenced tomato, and the results showed that both leaves and fruits exhibited larger lesion diameters compared to those of the wild type (Fig. 6F and G). These findings collectively suggested that *SlOpaque2* plays a crucial role in contributing to disease resistance against *B. cinereal* in tomatoes.

### SlCOP1-1-mediated stability of *SlOpaque2* enhances plant resistance to *B*. *cinerea*

**Figure 7.**
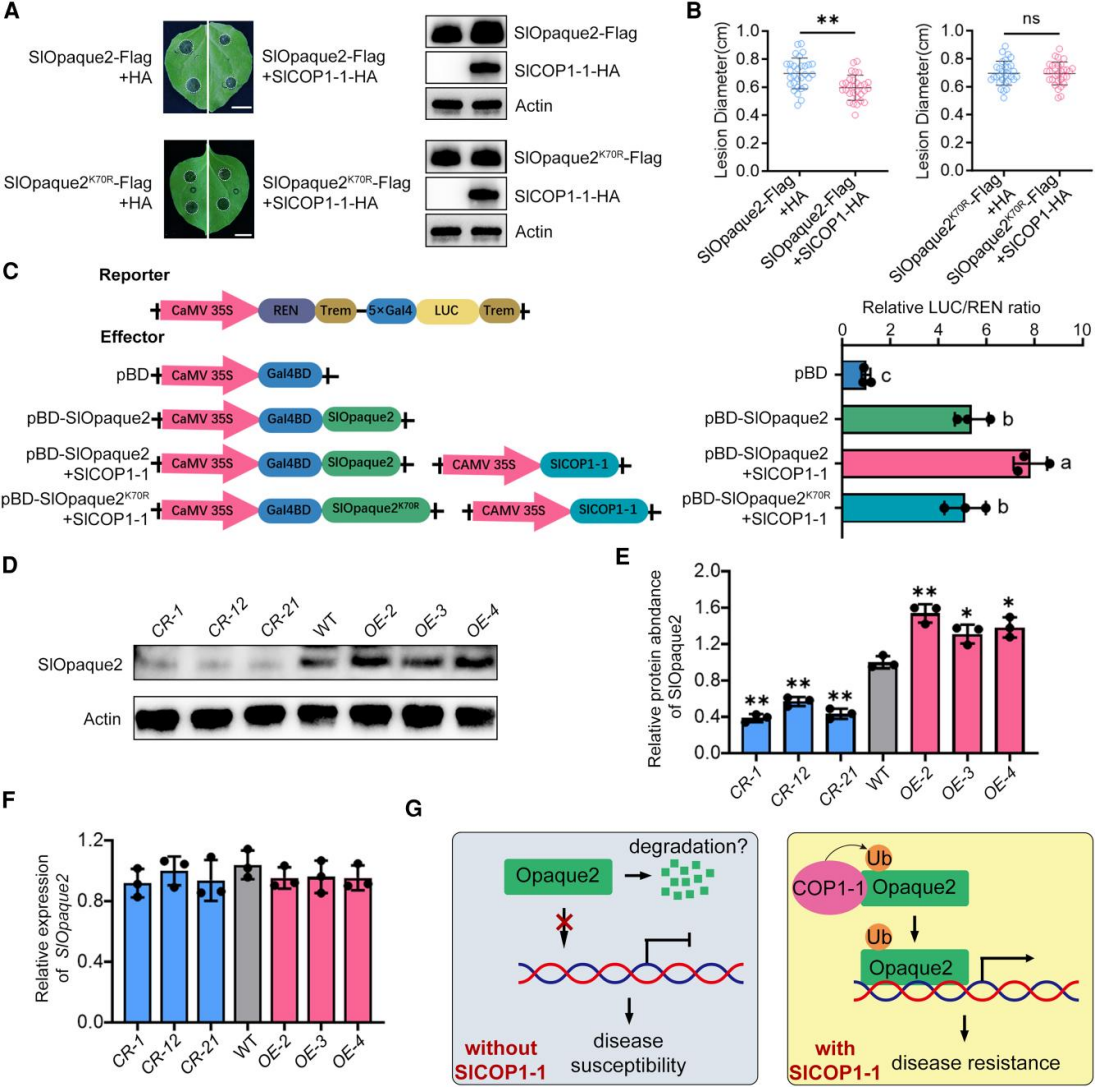
SlCOP1-1-mediated stability of SlOpaque2 enhances plant resistance to *Botrytis cinerea*. **A)**, **B)** Disease symptom A) and lesion diameters B) in *N. benthamiana* leaves overexpressing SlOpaque2-Flag and SlCOP1-1-HA (upper) or SlOpaque2^K70R^-flag and SlCOP1-1-HA (lower), followed by inoculation with *B. cinerea* for 3 d. For A), protein accumulation was analyzed by immunoblotting using anti-HA or anti-Flag antibodies, with actin serving as the loading control. For B), values represent means ± standard deviation (SD) (*n* = 30 inoculation sites in 15 leaves). Asterisks indicate statistically significant differences (*, *P* < 0.05, **, *P* < 0.01, Student's *t-*test). ns, not significant. **C)** Determination of SlOpaque2 transcriptional activity in *N. benthamiana* leaves. SlOpaque2 or SlOpaque2^K70R^ fused with GALBD protein (BD) and co-expressed with SlCOP1-1 were conformed with the dual-luciferase reporters (firefly luciferase, LUC; and renilla luciferase, REN) driven by 5 × GAL4 and 35S, respectively, in *N. benthamiana* leaves. Transcription activity is expressed by the ratio of LUC to REN activity. Values represent means ± SD of 3 independent experiments. ANOVA was used to analyze the data, with letters indicating statistically significant differences (*, *P* < 0.05, Tukey's test). **D)** Protein levels of SlOpaque2 in the fruit of wild-type, *Slcop1-1* mutants, and *SlCOP1-1* OE lines. Total proteins were extracted and subjected to immunoblotting analysis using SlOpaque2 antibody. Actin was used as the loading control. **E)** Quantification of the immunoblot bands in D) by Image J software. **F)** Gene expression of *SlOpaque2* in fruit of wild-type, *Slcop1-1* mutants and *SlCOP1-1* OE lines, as determined by RT-qPCR. *Actin* served as an internal control. In E) and F), values represent means ± SD of 3 independent experiments. Asterisks denote statistically significant differences (*, *P* < 0.05, **, *P* < 0.01, Student's *t*-test). **G)** The working model for the mechanism of SlCOP1-1 in the regulation of fruit resistance to *B. cinerea* through modulating SlOpaque2 ubiquitination and stability. In the absence of SlCOP1-1, SlOpaque2 degrades quickly. In the presence of SlCOP1-1, SlCOP1-1 mono-ubiquitinates SlOpaque2 and stabilizes its protein level, resulting in increased transcriptional activity of SlOpaque2 and enhanced resistance to *B. cinerea*.

Next, we investigated the impact of SlCOP1-1-mediated stability on SlOpaque2 in regulating resistance to *B. cinerea* using the *N. benthamiana* expression system. SlOpaque2-Flag was transiently co-expressed with SlCOP1-1-HA in *N. benthamiana* leaves, and *B. cinerea* was inoculated at 24 h post-agroinfiltration when the protein was evidently expressed (Supplementary Fig. S9). As shown in Fig. 7A and B, leaves co-expressing SlOpaque2 and SlCOP1-1 exhibited reduced disease severity compared to leaves expressing SlOpaque2 alone. Conversely, co-expression of the SlOpaque2^k70R^-Flag variant with SlCOP1-1 did not alleviate the disease symptoms of leaves. These data suggest that SlCOP1-1-mediated ubiquitination and stability enhance the ability of SlOpaque2 in plant resistance to *B. cinerea*.

To decipher the potential mechanism of SlCOP1-1 promoting SlOpaque2 function, we investigated whether the transcriptional activity of SlOpaque2 is regulated by SlCOP1-1. Using a dual reporter system in *N. benthamiana*, SlOpaque2 fused with GAL4BD (BD) was co-expressed with firefly luciferase (LUC) driven by 5 × GAL4 in combination with the minimal TATA region of CaMV35S, as well as renilla luciferase (REN) driven byCaMV35S. As shown in Fig. 7C, SlOpaque2 alone can activate the transcription of LUC. Co-expression of SlOpaque2 and SlCOP1-1 significantly enhanced the LUC transcription (*P* < 0.05, Turkey's *t*-test), while co-expression of the SlOpaque2^K70R^-flag variant and SlCOP1-1 showed no obvious effect on LUC transcription. This suggested that the transcriptional activity of SlOpaque2 is increased by SlCOP1-1-mediated ubiquitination and stability.

To further confirm the regulatory relationship of SlCOP1-1 with SlOpaque2 in tomato fruit, we examined the expression of SlOpaque2 in tomato fruit of *Slcop1-1* mutants, *SlCOP1-1* OE lines, as well as the wild type. Fruits harvested at 33 dpa were subjected to RT-qPCR analysis and immunoblotting analysis. The results showed that SlOpaque2 protein levels showed about 2-fold reduction in *Slcop1-1* mutant and 1.5-fold increase in *SlCOP1-1* OE lines, respectively (Fig. 7D and E). In contrast, no significant changes (*P* < 0.05, Student's *t*-test) occurred in *SlOpaque2* transcript levels between *Slcop1-1* mutants or *SlCOP1-1* OE lines and the wild type (Fig. 7F). These data indicated that SlCOP1-1 regulates the steady-state level of SlOpaque2 post-translationally in tomato fruit, which might, in turn, affect fruit resistance to *B. cinerea*. Based on these results, we propose a model for the regulation of tomato resistance to *B. cinerea* by SlCOP1-1-mediated SlOpaque2 mono-ubiquitination and stability (Fig. 7G).

## Discussion

### SlCOP1-1 orchestrates tomato fruit defense against *B. cinerea* while having minor influence on ripening

Although several studies have demonstrated that COP1 can regulate fruit ripening by controlling fruit pigmentation (Liu et al. 2004; Li et al. 2012; Liu et al. 2023, Zhao et al. 2023), SlCOP1-1 seemingly exerts only a minor influence on this process. In our study, we generated knockout mutants and OE lines of *SlCOP1-1* through stable genetic transformation and systematically observed their ripening characteristics, including the days from anthesis to the breaker stage, fruit color, and global protein changes. Unlike transiently transformed fruit in prior studies, mutant fruits from our stable genetic transformation provide a more conducive material to observe the entire ripening progress. We found significant, yet minor changes (*P* < 0.05, Student's *t*-test) in the days to the breaker stage of *Slcop1-1 mutants* or *SlCOP1-1* OE lines compared to wild-type fruit, and only a few ripening-related proteins or genes were differentially identified in these fruits (Fig. 1; Supplementary Fig. S5; Supplementary Data Set 1). These results suggest that SlCOP1-1 play a minor role in controlling fruit ripening progress. Considering that *LeCOP1like*, another homologous gene to *AtCOP1* in tomatoes, have been reported to regulate fruit ripening through its negative regulatory role in pigmentation (Liu et al. 2004), we infer that COP1 genes in tomatoes have undergone apparent functional divergence following gene duplication. Additionally, previous studies have shown that OE of *Solanum melongena* COP1 in tomatoes can affect fruit ripening through influencing ethylene signaling (Naeem et al. 2019). Further research could elucidate whether SlCOP1-1 or LeCOP1like regulates fruit ripening through the ethylene pathway.

While COP1 has been previously implicated in plant resistance against viruses and bacteria (Jeong et al. 2010; Gangappa 2018; Lim et al. 2018), its role in regulating disease resistance to fungal pathogen remains poorly understood. Our study found that *SlCOP1-1* is a *B. cinerea* responsive gene, with its expression significantly increasing (*P* < 0.05, Student's *t*-test) in tomato fruit following *Botrytis* inoculation (Supplementary Fig. S10). Furthermore, we provided evidence that COP1 can positively regulate fruit resistance to *B. cinerea* (Fig. 2). Additionally, we proposed that the SlCOP1-1-SlOpaque2 module contributes to mediating this process, which not only enriches the pleiotropic functions of COP1 but also provides insights into understanding the gene regulatory networks contributing to fruit disease resistance. Nevertheless, it cannot be ignored that there may be other mechanisms through which SlCOP1-1 regulates fruit resistance. Indeed, our study revealed a substantial number of differently expressed proteins associated with disease resistance in *Slcop1-1* mutant and SlCOP1-1 OE line (Fig. 3; Supplementary Data Set 1). Some of these proteins have been reported to be involved in fruit resistance to fungal pathogens, such as those related to cell wall metabolism [e.g. xyloglucan endotransglucosylase/hydrolase, XTH (Miedes 2007)], the redox system (e.g. catalase, CAT; peroxidase, POD; Peptide methionine sulfoxide reductase, MsrA) (Hua et al. 2018; Camejo et al. 2019), heat shock proteins [e.g. HSF8 (Yang et al. 2023)], and other resistant proteins [e.g. carbonic anhydrase, CA (Zhou et al. 2023]. Therefore, it is worthwhile to further explore whether there are direct regulatory relationships between COP1 and these resistance pathways.

### SlOpaque2 is a multifunctional transcription factor regulated by multiple levels

Opaque2, identified as a bZIP transcription factor in maize, has been well characterized for its control of various maize agronomic traits, including kennel nutrients, soft texture, and susceptibility to disease (Loesch et al. 1976; Schmidt et al. 1990). Recent investigations on the transcriptional regulatory framework of Opaque2 have unveiled its diverse functions in directly and indirectly regulating genes associated with nutrient accumulation, nitrogen metabolism, and stress resistance (Li et al. 2015). Although the role of Opaque2 is well-established in maize, its function in other plants, including tomato, remains uncertain. In our study, we observed the obvious transactivation activity of SlOpaque2 and its upregulated expression during fruit ripening (Fig. 6). By using in vivo incubation assays, we established a negative association between Opaque2 and decay caused by *B. cinerea* (Fig. 6), suggesting a role for SlOpaque2 in fruit resistance to *B. cinerea*. In maize, ZmOpaque2 imparts plant resistance, possibly through directly regulating the expression of antifungal proteins (e.g. ribosome-inactivating protein 1, RIP1), stress resistance-related protein (e.g. lactoylglutathione lyase, LGL), and endopeptidase inhibitors (Lohmer et al. 1991; Li et al. 2015). However, these target proteins of Opaque2 appear not to exist or are not altered in *Slcop1-1 mutant* or *SlCOP1-1* OE lines (Supplementary Data Set 1), implying a distinct target network modulated by SlOpaque2 in tomato, which requires further investigation.

Opaque2 is subject to regulation at multiple levels. Previous studies demonstrated that maize Opaque2 activity is regulated by RNA abundance, protein phosphorylation, and protein poly-ubiquitination during its diurnal regulation of nutrient accumulation (Ciceri et al. 1999; Li et al. 2020). Recently, the maize Sucrose non-fermenting-1 Related Protein Kinase 1 (SnRK1)-RING Finger Protein with WD40 Domain 3 (RFWD3)-Opaque2 signaling axis was identified as modulating ZmOpaque2 activity in response to sucrose levels, in which Opaque2 is oligo-ubiquitinated by ZmRFWD3 E3 ligase, enhancing Opaque2 nuclear localization ability (Li et al. 2020). In our study, we observed ubiquitination of SlOpaque2 and SlOaque2 degradation mediated by the ubiquitin-proteosome system (Fig. 5), indicating a similar regulatory mechanism in different plant systems. Furthermore, we found that the ubiquitination of SlOpaque2 at the K70 site, catalyzed by SlCOP1-1 E3 ligase, reduces poly-ubiquitination and degradation of SlOpaque2 protein (Fig. 5). This suggests that SlCOP1-1 may compete with other E3 ligases responsible for SlOpaque2 poly-ubiquitination, binding to and then mono-ubiquitinating SlOpaque2, as the case of COP1 and Anaphase Promoting Complex (APC) E3 ligase in the stability of double-stranded RNA-binding protein 4 (DRB4) during plant resistance to viruses (Lim et al. 2018). Given that the oligo-ubiquitination of ZmOpaque2 enhances its nuclear localization ability (Li et al. 2020), it would be interesting to explore whether mono-ubiquitination mediates the shuttle ability of SlOpaque2, thereby avoiding its UPS-mediated degradation.

### SlCOP1-1-SlOpaque2 module represents a mechanism for enhancing fruit disease resistance

COP1 typically plays a role in protein degradation, controlling various biological processes by directly or indirectly degrading the regulators in plants (Shi et al. 2016). However, several cases showed that COP1 deviates from its canonical degradation role. For instance, COP1/Suppressor of PhyA (SPA) promotes the stability of PHYTOCHROME INTERACTING FACTOR 3 (PIF3), a key repressor of photomorphogenesis, by blocking the interaction of PIF3 with its kinase Brassinosteroid-Insensitive 2 (BIN2), contributing to the precise regulation of skotomorphogenesis (Ling et al. 2017). In our study, we unveil another noncanonical mechanism of COP1 in plants, by which SlCOP1-1 mediates the mono-ubiquitination and stability of SlOpaqure2, thereby enhancing SlOpaque2 transactivation activity and fruit resistance to *B. cinerea*. Thus, the SlCOP1-1-Opaque2 module represents a mechanism for COP1 to mediate disease resistance. Nevertheless, there is much to be elucidated about the precise regulation of the SlCOP1-1-SlOpaque2 module in future studies. Specifically, it is unknown whether light signals regulate the SlCOP1-1-SlOpaque2 module. Native SlCOP1-1 in tomato fruit were more abundant in the fruit nucleus under dark conditions and in fruit non-nuclear cellular components under light conditions (Supplementary Fig. S11), suggesting that the localization of SlCOP1-1 in fruit is light-induced, and the interaction between SlCOP1-1 and SlOpaque2 might primarily occur in darkness in fruit. Our study indeed revealed that several differentially expressed proteins responding to high or low light intensity were significantly enriched (*P* < 0.05, background-based *t*-test) in *Slcop1-1* mutant and *SlCOP1-1* OE fruits (Supplementary Data Set 2). Given that COP1 is a key component of the light signaling pathway (Deng et al. 1992) and Opaque2 appears to be regulated by the day–night rhythm (Ciceri et al. 1999; Li et al. 2020), further research is needed to unveil the role of light signals in the regulation of the SlCOP1-1-SlOpaque2 module. This will aid in understanding the relationship between light and fruit resistance to pathogens.

As tomato fruit ripens, the expression of SlCOP1-1 and SlOpaque2 increases, and the SlCOP1-1-SlOpaque2 module-mediated fruit disease resistance is also enhanced. However, ripening fruit becomes more susceptible to inoculation by *B. cinerea* (Prusky 1996). One possible explanation for this contradiction is that as the fruit ripens, the texture softens, sugars, acid, and water accumulate, and the levels of resistant substances decrease, which facilitates pathogen invasion and multiplication (Cantu et al. 2008; Alkan et al. 2015). If the disease susceptibility caused by these factors in fruit becomes stronger than the fruit's inherent disease resistance, the fruit will exhibit susceptibility to disease. Conversely, if the disease resistance pathway of the fruit is strengthened, the ability of the fruit to defend against pathogen fruit will be enhanced. In our study, tomato fruits overexpressing *SlCOP1-1* exhibited substantially stronger disease resistance than wild-type fruit and the *Slcop1-1* knockout fruits; this suggests that the defense mediated by the SlCOP1-1-SlOpaque2 module surpasses the effect of SlCOP1-1 in accelerating fruit ripening. Therefore, the SlCOP1-1-SlOpaque2 module represents potential target genes for improving fruit disease resistance through genetic breeding in the future.

## Materials and methods

### Plant materials

Tomato plants (*Solanum lycopersicum* cv. Ailsa Craig) were grown in a greenhouse under standard culture conditions, with regular fertilizer and supplementary lighting. To accurately assess the fruit ripening stage, flowers were labeled at anthesis. Both transgenic and wild-type fruits were harvested at 20-, 36-, 39-, 42-, and 45 dpa, corresponding to the immature (IM), mature green (MG), breaker (Br), orange (Or), and red ripe (RR) stages of wild-type fruits, respectively. *Nicotiana benthamiana* and tomato cv. Micro-Tom plants were cultivated in a growth room maintained at 22°C, with 60% to 80% relative humidity and a 16/8 h light/dark photoperiod. Leaves or fruits were immediately collected, frozen in liquid nitrogen, and stored at −80°C for subsequent analysis.

### Phylogenetic analysis

The amino acid sequences of SlCOP1-1 (Solyc12g005950), SlCOP1-2 (Solyc11g011980), and LeCOP1LIKE (Solyc11g005190) were acquired from Sol Genomics Network (SGN; https://solgenomics.net/tools/blast/). Additional COP1 homologous proteins from different species were obtained from the National Center for Biotechnology Information (NCBI) database (https://www.ncbi.nlm.nih.gov/). Multiple protein sequence alignments were performed using DNAMAN software (version 8) with default parameters. The phylogenetic tree was generated by MEGA (version 10.1.8) with bootstrap values from 500 replicates for each branch.

### RT-qPCR analysis

Total RNA was separately extracted from tomato pericarps and other tissues using the hot phenol method (Moore et al. 2005). Genomic DNA digestion, first-strand cDNA synthesis, and RT-qPCR were performed according to previously described methods (Wang et al. 2020). The 2^−ΔΔCt^ method was used to calculate relative gene expression levels, with *Actin* (Solyc11g005330) serving as the normalization control across diverse samples. Primer sequences for PCR amplifications are listed in Supplementary Table S2. Each experiment contained 3 independent biological replicates, with each replicate consisting of a pool of 5 tissues collected from at least 3 plants.

### Subcellular localization

For subcellular localization analysis, the CDS of *SlCOP1-1* and *SlOpaque2* were individually cloned into the pCambia2300-eGFP and pCambia2300-mCherry vectors, resulting in the generation of OE constructs *SlCOP1-1-eGFP* and *SlOpaque2-mCherry*. The constructed plastids were then introduced into *Agrobacterium tumefaciens* strain GV3101, which were subsequently infiltrated into *N. benthamiana* leaves according to the method described by Sparkes et al. (2006). For colocalization analysis, *A*. *tumefaciens* strain GV3101 harboring the constructs *SlCOP1-1-eGFP* and *SlOpaque2-mCherry* were co-infiltrated into *N*. *benthamiana* leaves. Following infiltration, the *N*. *benthamiana* plants were cultured for 36 h. Mesophyll protoplasts were isolated according to the previously described method (Lei et al. 2014) and visualized under a Leica confocal microscope (Leica DMI600CS).

GFP fluorescence was excited with a 488-nm laser and monitored at 505 to 550 nm, while mCherry was excited at 561 nm and monitored at 610 to 650 nm, both with a pinhole setting of 1.5 AU.

### Polyclonal antibodies preparation

Polyclonal antibodies were produced by Abmart Shanghai Co., Ltd. (China), following the protocol described by Wang et al. (2017). For the preparation of the SlCOP1-1-specific antibody, the immune peptide RRMGDKEEGGSV was synthesized and employed. For the preparation of the SlOpaque2-specific antibody, a fragment of SlOpaque2 lacking the conserved domain was expressed, purified from *Escherichia coli* BL21 (DE3), and used as an antigen protein. Rabbits were immunized with either the SlCOP1-1 synthetic peptide or the SlOpaque2 protein antigen to produce immune serum. The obtained sera were then subjected to affinity-purification using the corresponding synthetic peptide or proteins. The primers used for the amplification of the SlOpaque2 fragment are listed in Supplementary Table S2.

### Protein extraction and immunoblot analysis

Total protein extraction from tomato fruit and *N*. *benthamiana* leaves was performed according to a previously established protocol (Wang et al. 2020). Nuclear isolation and nuclear protein extraction were conducted followed by the previous method (Wang et al. 2021). Each extraction was conducted with 3 independent biological replicates, each consisting of a pool of 5 leaves or fruits collected from at least 3 plants. Immunoblot analysis was conducted following a previously described method (Wang et al. 2020), with actin serving as an internal control. In brief, protein samples were separated by 10% (w/v) SDS-PAGE and then transferred to a PVDF membrane (Millipore, IPVH00010) using a semi-dry transblotter unit (Bio-Rad, USA). The membranes were blocked for 1 h at room temperature with 5% (w/v) nonfat milk or 1% (w/v) BSA in TBST buffer. Immunoblotting was carried out with the corresponding primary and secondary antibodies at room temperature for 1 h. The immunoreactive bands were visualized using a chemiluminescence detection kit (Mei5 Biotechnology Co., Ltd, China) following the manufacturer's protocol.

### Plant transformation

For the construction of the CRISPR/Cas9 vector, 2 specific sgRNA targeting the coding region of SlCOP1-1 were designed and individually incorporated into the pYLCRISPR/Cas9Pubi-H binary plasmid, following the protocol described by Ma et al. (2015). The off-target sites were predicted using CRIPR-P (version 2.0, http://crispr.hzau.edu.cn/CRISPR2/). To generate the 35S:*SlCOP1-1* construct, the CDS of *SlCOP1-1* was inserted into pCambia1302 vector between CaMV 35S promoter and NOS terminator. Following verification through sequencing, the resulting constructs were transformed into *A. tumefaciens* strain GV3101, which were then used for tomato transformation following a previously established method (Fillatti et al. 1987). The CRISPR/Cas9-based knockout mutants were screened at the targeted sites using PCR amplification and sequencing. Transgenic OE plants were confirmed by PCR genotyping. The primers used for vector construction and screening the transgenic plants are listed in Supplementary Table S2.

### Pathogen inoculation

Inoculation of fruits and leaves with *B*. *cinerea* was performed according to the procedures described in a previous study (Zhou et al. 2023). The *B*. *cinerea* strain B05.10 was cultured on Potato Dextrose Agar plates for 2 wk. Spores were collected and adjusted to a final concentration of 1 × 10^5^ conidia per milliliter. Five microliters of conidia suspension were then inoculated into each pre-wounded fruit or detached leaves. The inoculated fruit or leaves were kept in a growth room at 22°C, with 60% to 80% relative humidity and a 16/8 h light/dark photoperiod. Fungal growth was evaluated by quantifying the ratio of *B*. *cinerea Actin* relative to tomato *Actin* through qPCR amplification using DNA extracted from lesion tissue. The primers used for qPCR are listed in Supplementary Table S2. Each experiment was conducted with 3 independent biological replicates, with each replicate consisting of 15 fruits or leaves, and each fruit or leaf providing 2 to 4 inoculation sites.

### Quantitative proteomic analysis

Quantitative proteomic analysis was performed according to a previously established method (Wang et al. 2023). Proteins were separately extracted from 2 *Slcop1-1* mutant lines (*CR-1*, *CR-21*), 2 *SlCOP1-1* OE lines (*OE-2*, *OE-3*), and the wild-type fruits. About 100 *μ*g of proteins from each sample were subjected to reduction, alkylation, and tryptic digestion. The resulting tryptic peptides were labeled using the TMT reagents 6-plex Kit (Thermo Scientific) following the manufacturer's protocol. The TMT-labeled peptides were then combined, lyophilized, and subjected to high-pH reversed-phase chromatography. A total of 24 fractions were collected, consolidated into six pools, and subsequently desalted before LC-MS/MS analysis. Three independent biological replicates were applied for proteomic analysis, with each replicate using 10 fruits collected from at least 3 plants.

Protein identification and relative quantification were conducted using Proteome Discoverer software (version 2.4). Mass spectra data were utilized for searching the tomato protein database (tomato build_SL3.0 reference genome; https://solgenomics.net/ftp/tomato_genome/assembly/build_3.00/). A reverse database search strategy was employed to determine the global False Discovery Rate (FDR) for peptide identification. Background-based *t*-tests were applied to assess statistically significant differences in protein levels. Proteins meeting the criterion of FDR <0.01, along with fold change ratios >1.5 or <0.67 (*P* < 0.05), were considered statistically significant. GO enrichment analysis was performed based on the EggNOG database using eggnog-mapper software (v2.0). The enrichment of differentially expressed protein was assessed using a 2-tailed Fisher's exact test.

### Y2H analysis

Y2H screening was conducted following a previously described method (Wang et al. 2017). A tomato fruit cDNA library, constructed in the prey vector pGADT7 (AD), was screened with the intact CDS of *SlCOP1-1* cloned into pGBKT7 (BD) in yeast (*Saccharomyces cerevisiae*) strain AH109 (Clontech), following the manufacture's manual (Clontech). For Y2H analysis of SlCOP1-1 with other proteins, the intact CDS of these proteins were individually cloned into the AD vector. The resulting recombinant vectors were co-transformed with BD-SlCOP1-1 into *S*. *cerevisiae* strain AH109 and then cultured on selective media, including SD/-Leu/-Trp media (-LW), SD/-Leu/-Trp/-His media (-LWH), and SD/-Leu/-Trp/-His/-Ade media (-LWHA), with or without X-α-gal. BD and AD, BD and AD-SlOpaque2, as well as BD-SlCOP1-1 and AD, were co-transformed in parallel as negative controls. The primers used in vector construction are listed in Supplementary Table S2.

### LCI assay

The LCI assay was conducted following the method described by Chen et al. (2008). The CDS of *SlCOP1-1* and *SlOpaque2* were cloned into split LUC vectors, pCambia1300-nLUC/cLUC, to generate 35S:*SlCOP1-1-nLUC* and 35S:*cLUC-SlOpaque2*, respectively. The recombinant constructs were transformed into *A*. *tumefaciens* strain GV3101, which were then infiltrated into *N*. *benthamiana* leaves. After a 2-d incubation, the infiltrated leaves were sprayed with luciferin substrate and kept in the dark for 5 min. The experiment was performed with at least 3 *N*. *benthamiana* leaves. Chemiluminescence Imaging System (Tanon) was applied for image capture. The primers used in vector construction are listed in Supplementary Table S2.

### Pull-down assay

The pull-down assay was conducted according to a previously described protocol (Wang et al. 2020). The CDS of *SlCOP1-1* and *SlOpaque2* were individually cloned into pETMALc-H and pGEX-4T-2 vectors (GE Healthcare, USA) and introduced into competent *E*. *coli* strain Rosetta (DE3) cells (TransGen Biotech, China) for prokaryotic expression. The recombinant GST-SlOpaque2 and MBP-SlCOP1-1 proteins were purified separately using glutathione Sepharose beads (GE Healthcare, USA) and amylose resin (New England Bio-labs, USA) following the respective user manual. The purified GST-SlCOP1-1 and MBP-SlOpaque2 proteins were mixed and then collected using anti-HA agarose (Cell Signaling Technology). The proteins were then eluted and subjected to immunoblotting analysis using anti-GST or anti-MBP antibodies. The primers used in vector construction are listed in Supplementary Table S2.

### Co-IP assay

The Co-IP assay was conducted following a previously established protocol (Wang et al. 2020). The CDS of *SlCOP1-1* and *SlOpaque2* were individually cloned into the pCambia2300 vector to generate 35S:*SlCOP1-1-HA* and 35S:*SlOpaque2-GFP* plasmids. The constructed plasmids were then transformed into *A*. *tumefaciens* strain GV3101, which were subsequently co-infiltrated into *N*. *benthamiana* leaves. After 48 h of infiltration, total leaf proteins were extracted and inoculated with anti-HA magnetic beads (Cell Signaling Technology) at 4°C for 2 h. Following collection, washing, and elution, the proteins eluted from magnetic beads were subjected to immunoblotting analysis using anti-HA or anti-GFP antibodies (Cell Signaling Technology). A negative control was established using mCherry-HA. The primers used in vector construction are listed in Supplementary Table S2.

### In vitro ubiquitination assay

The in vitro ubiquitination assay was conducted following the protocol described by Xie et al. (2002). For determining the E3 ligase activity of SlCOP1-1, MBP-SlCOP1-1 expressed and purified from *E. coli*, as described above, was incubated with His-tagged wheat E1 (UBA1, M55604.1), human E2 (UBCh5b, U39317.1), and Arabidopsis ubiquitin (UBQ14, At4g02890), provided by professor Jingbo Jin (Institute of Botany, Chinese Academy of Sciences). For detecting the ubiquitination of SlOpaque2 by SlCOP1-1, the CDS of *SlOpaque2* was inserted into pET-30a and expressed in *E. coli* as the recombinant S-tagged SlOpaque2 protein. Following purification, the S-SlOpaque2 protein was added to the above in vitro ubiquitination assay. The resulting reaction products were analyzed via immunoblot using anti-S-Tag, anti-MBP or anti-Ub antibodies (Cell Signaling Technology). The primers used in vector construction are listed in Supplementary Table S2.

### In vivo ubiquitination assay

The in vivo ubiquitination assay using *N*. *benthamiana* transient expression system was conducted following the protocol described by Wang et al. (2020). The CDS of *SlCOP1-1* and *SlOpaque2* was separately cloned into pCambia1302 vector to construct 35S:*SlCOP1-1* and 35S:*SlOpaque2-HA* plasmids. The resulting constructs were co-transformed into *A*. *tumefaciens* strain GV3101, followed by *A*. *tumefaciens* mediated infiltration of *N*. *benthamiana* leaves. After 36 h of infiltration, total proteins from *N*. *benthamiana* leaves were extracted and then subjected to incubation with anti-HA magnetic beads (Cell Signaling Technology). To detect the ubiquitination of SlOpaque2 in tomato fruit, total proteins extracted from fruits of *SlCOP1-1* OE lines and *Slcop1-1* mutants were immunoprecipitated with anti-SlOpaque2 antibody. Following collection and washing, the immunoprecipitated proteins were eluted from beads and analyzed by immunoblot using anti-HA or anti-Ub antibodies (Cell Signaling Technology). The primers used in vector construction are listed in Supplementary Table S2.

### Protein stability and degradation assays

Protein stability and degradation assays were conducted following the protocol described by Wang et al. (2020). The CDS of *SlOpaque2* was cloned into the pCambia1302 vector to construct 35S:*SlOpaque2-Flag* vectors. The 35S:*SlCOP1-1-HA* was constructed as described above. The resulting plasmids were transformed into *A*. *tumefaciens* strain GV3101, which were then co-infiltrated in *N*. *benthamiana* leaves. For protein stability assay, the leaves were treated with 50 *μ*M MG132 after 36 h of infiltration and sampled 2 h after treatment. For monitoring the degradation rate, the leaves were treated with 250 *μ*M CHX 36 h after infiltration and then incubated for another 2 h before sampling. After total protein extraction from *N*. *benthamiana* leaves, immunoblot analysis was conducted using anti-flag (Cell Signaling Technology). The intensity of protein bands was quantified using ImageJ software (https://imagej.net/ij/index.html), with the quantitative values representing the means of 3 biological replicates. The primers used in vector construction are listed in Supplementary Table S2.

### VIGS assay

The VIGS assay was conducted following the protocol described by Fu et al. (2005) with minor modifications. A specific cDNA fragment of *SlOpaque2* was integrated into the pTRV2 vector to generate pTR2-*SlOpaque2*, which was then introduced into *A*. *tumefaciens* strain GV3101. Equivalent aliquots of *A*. *tumefaciens* containing pTR2-*SlOpaque2* or pTRV1 were mixed, collected, and resuspended in infiltration media to achieve an optical density of 0.3 at OD_600_. Approximately 20 Micro-Tom tomato plants (4-wk-old) were subjected to needle injection into the peduncles. The primers used in vector construction are listed in Supplementary Table S2.

### Transcriptional activity assay

The transcriptional activity assay in *N. benthamiana* leaves was conducted following the previously described method (Wang et al. 2021). Briefly, the CDS of *SlOpaque2* was cloned into the effector plasmid (pEAQ-BD), fusing with GAL4BD riven by the CaMV35S. The effector construct was co-transformed with a dual LUC reporter vector into *N. benthamiana* leaves using *A. tumefaciens* strain GV3101. The reporter vector contained REN driven by CaMV35S and firefly LUC fused with 5 × GAL4 under the control of the minimal TATA region of CaMV35S. Following 36 h of incubation, LUC and REN luciferase activities were assessed using dual-luciferase assay kits (Promega), and the transcriptional activity was quantified by the ratio of LUC to REN. The primers used in vector construction are listed in Supplementary Table S2.

### Statistical analyses

GraphPad Prism 10.0 was used for statistical analysis. Data are shown as the means ± standard deviation (SD) of at least 3 independent biological experiments. Statistical significance was analyzed by 2-tailed Student's *t*-test. For comparisons among multiple groups, ANOVA was performed. *P* < 0.05 was considered statistically significant, with individual *P*-values indicated by asterisks in figures (*, *P* < 0.05; **, *P* < 0.01; ***, and *P* < 0.001).

## Accession numbers

Sequence data from this article can be found in Supplementary Table S2. The mass spectrometry proteomics data have been deposited to the ProteomeXchange Consortium (https://www.iprox.cn/page/home.html) via the iProX partner repository with the dataset identifier IPX0009181000 and PXD053671.

## Supplementary Material

kiae404_Supplementary_Data

## Supplementary data

The following materials are available in the online version of this article.

**Supplementary Figure S1.** Phylogenetic analysis of 3 putative tomato COP1 orthologs.

**Supplementary Figure S2.** Off-target analysis in *Slcop1-1* mutants.

**Supplementary Figure S3.** Western blot analysis of SlCOP1-1 in *Slcop1-1* mutants and *SlCOP1-1* OE lines.

**Supplementary Figure S4.** Transcript levels of *SlCOP1-2* and *LeCOP1LIKE* in *Slcop1-1* mutants and *SlCOP1-1* OE lines.

**Supplementary Figure S5.** Ripening phenotype of *Slcop1-1* and *SlCOP1-1* OE fruits.

**Supplementary Figure S6.** Y2H validation confirms the interaction between SlCOP1-1 and candidates identified by Y2H screen.

**Supplementary Figure S7.** SlCOP1-1-mediated ubiquitination of SlOpaque2 in tomato.

**Supplementary Figure S8.** Fruit phenotype and ripening-related gene expression analysis in *SlOpaque2*-silenced fruits.

**Supplementary Figure S9.** Transient co-expression of SlOpaque2 and SlCOP1-1 in *Nicotiana benthamiana* leaves.

**Supplementary Figure S10.** *SlCOP1-1* expression in fruits with or without *B. cinerea* inoculation.

**Supplementary Figure S11.** Subcellular localization of SlCOP1-1 in tomato fruit cells under light and dark conditions.

**Supplementary Table S1.** Proteins identified via Y2H screen using SlCOP1-1 as a bait.

**Supplementary Table S2.** Primers used in this study.

**Supplementary Data Set 1.** TMT-based quantitative proteomic analysis of fruits of the wild-type, *Slcop1-1* mutant, and *SlCOP1-1* OE lines.

**Supplementary Data Set 2.** GO enrichment analysis of the differentially expressed proteins in the *Slcop1-1* mutant and *SlCOP1-1* OE lines compared to the wild type.
