# Supplementary material for: E3 ligase SlCOP1-1 stabilizes transcription factor SlOpaque2 and enhances fruit resistance to Botrytis cinerea in tomato
Source: Plant Physiol. 2024 Jul 30;196(2):1196–213. doi: 10.1093/plphys/kiae404 (PMC11444291; doi:10.1093/plphys/kiae404)
Supplement: kiae404_Supplementary_Data [file kiae404_supplementary_data.zip › Supplementary Data.pdf]

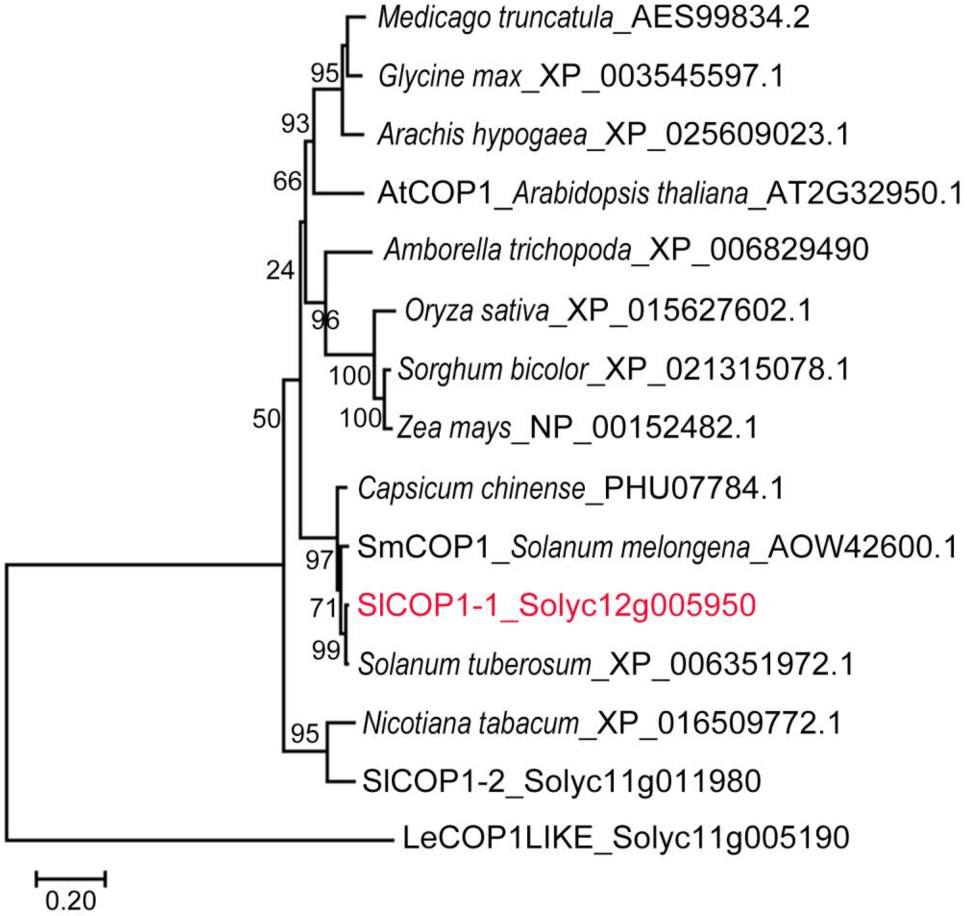

Supplementary Figure S1. Phylogenetic analysis of three putative tomato COP1 orthologs. The amino acid sequences of SlCOP1-1, SlCOP1-2, and LeCOP1LIKE were acquired from Sol Genomics Network (SGN; https://solgenomics.net/tools/blast/). Other COP1 homologous proteins from different species were obtained from the National Center for Biotechnology Information (NCBI) database (https://www.ncbi.nlm.nih.gov/). Multiple protein sequence alignments were performed using DNAMAN software (version 8) with default parameters. The phylogenetic tree was generated by MEGA (version 10.1.8) with bootstrap values from 500 replicates for each branch. SlCOP1-1 was labeled as red letters in the tree. The tree scale is 0.20.

Name	Target sequence	Putative off-target position	Sequence of the putative off-target site	Off-score	Region	Off-target
T1-Off1	GGAAAGTTCAGTTGGAGGGGTGG	SL2.5ch07: 67535285	TGAAAGTTCAGCAGGAGGAGTGG	0.468	CDS	No
T1-Off2	GGAAAGTTCAGTTGGAGGGGTGG	SL2.5ch05: 16799377	GGAAAGTTCACTTGGAGAAGAGG	0.212	Intron	No
T2-Off1	GGGGGGTAGTGTAACTCTAAGGG	SL2.5ch12: 66047752	AGGTGGAAGTGCAACTCTAAGGC	0.006	CDS	No

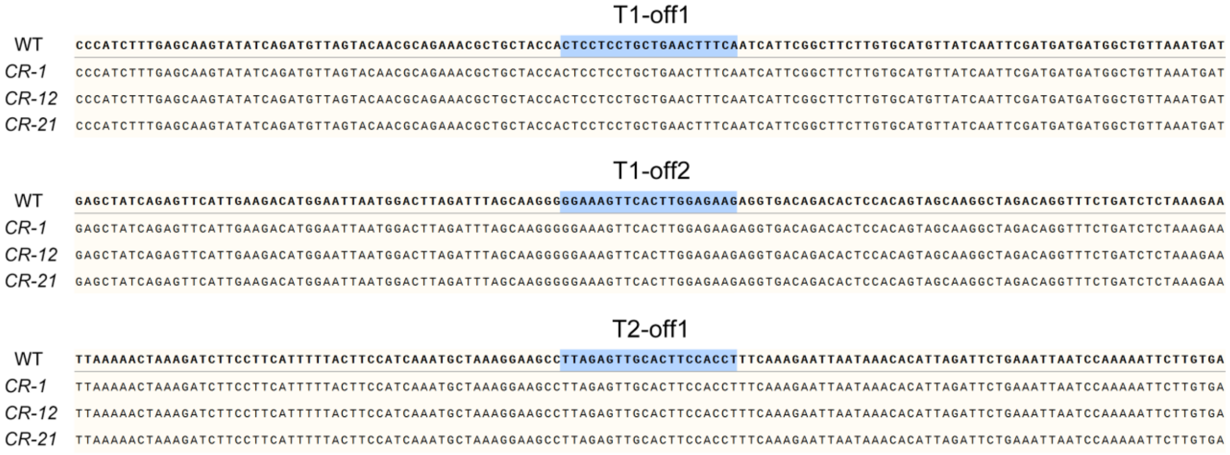

Supplementary Figure S2. Off-target analysis in *Slcop1-1* mutants. CRISPR-P (http://crispr.hzau.edu.cn/CRISPR/) was used to predict potential off-target sites for Target 1 (T1-Off) and Target 2 (T2-Off). We labeled the protospacer adjacent motif (PAM) as the underlined letters in column 'Target sequence', while the sequence difference between off-target sites and targets as the red letters in column 'Sequence of putative off-target site'. The potential off-target sites were amplified by PCR based on flanking these sites using genomic DNA as templates. The alignment of sequences surrounding putative off-target sites in the wild type (WT) and *Slcop1* mutants (*CR-1*, *CR-12*, and *CR-21*) is presented, with the potential off-target sites highlighted in blue.

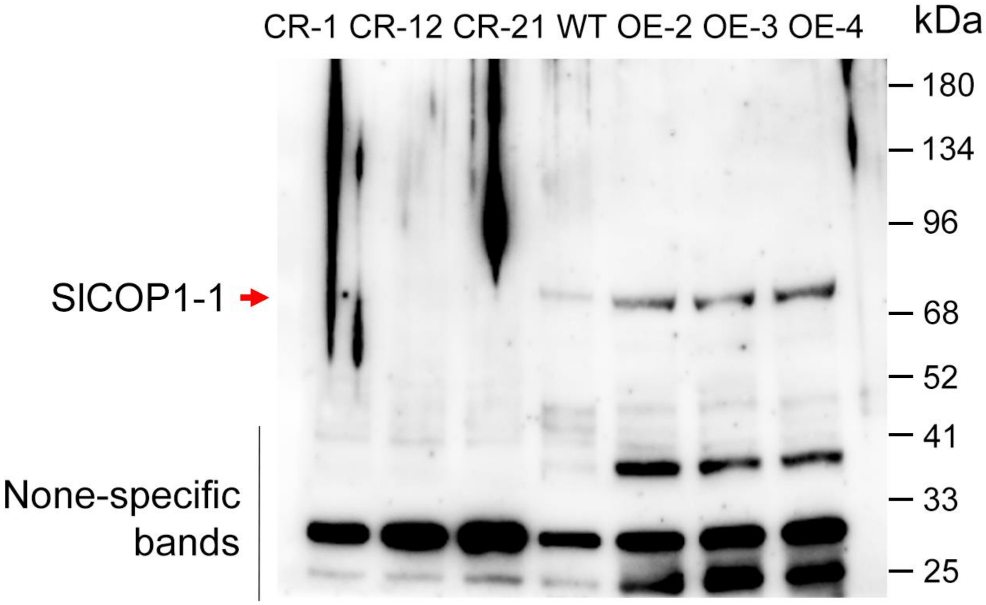

Supplementary Figure S3. Western blot analysis of SlCOP1-1 in *Slcop1-1* mutants and *SlCOP1-1* OE lines. Proteins extracted from fruits of *Slcop1-1* mutants (*CR-1*, *CR-12*, *CR-21*) and *SlCOP1-1* overexpression lines (*OE-2*, *OE-3*, *OE-4*) were immunoblotted with anti-SlCOP1-1 antibody. The red arrow indicates the specific SlCOP1-1 bands. Non-specific bands include putative SlCOP1-1 homologous proteins or SlCOP1-1 degradation products. kDa, kilodalton.

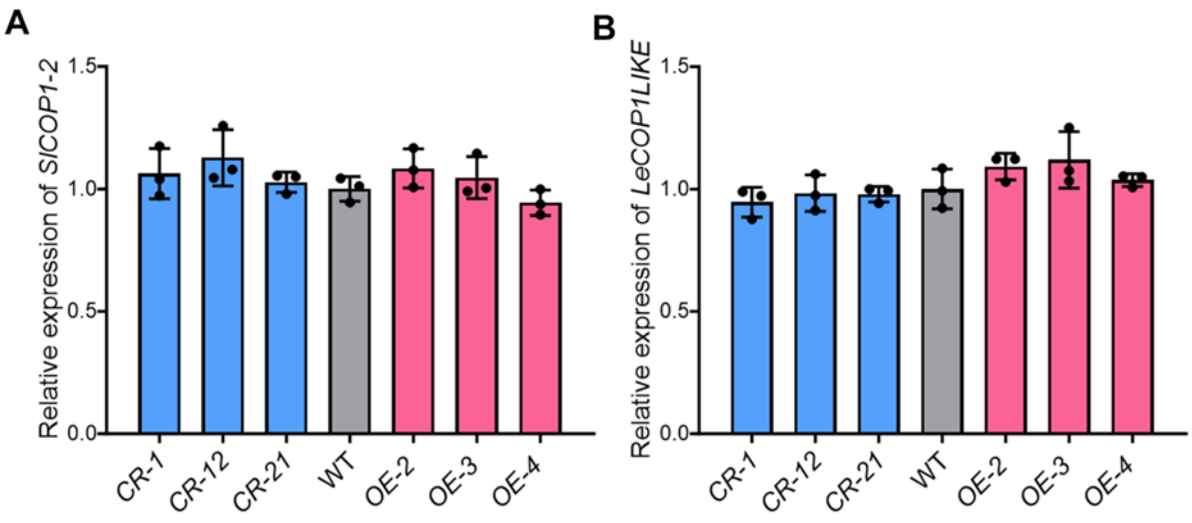

Supplementary Figure S4. Transcriptional levels of *SlCOP1-2* and *LeCOP1LIKE* in both *Slcop1-1* mutants and *SlCOP1-1* OE lines. RT-qPCR was performed to determine the changes of *SlCOP1-2* (A) and *LeCOP1LIKE* (B) in the transgenic plants compared to the wild-type plants (WT), with *Actin* used as an internal control. Values represent means ± standard deviation (SD) of three independent experiments. *CR-1*, *CR-12*, and *CR-21* represent three independent *Slcop1-1* mutants. *OE-2*, *OE-3*, and *OE-4* represent three independent *SlCOP1-1* overexpression (OE) lines.

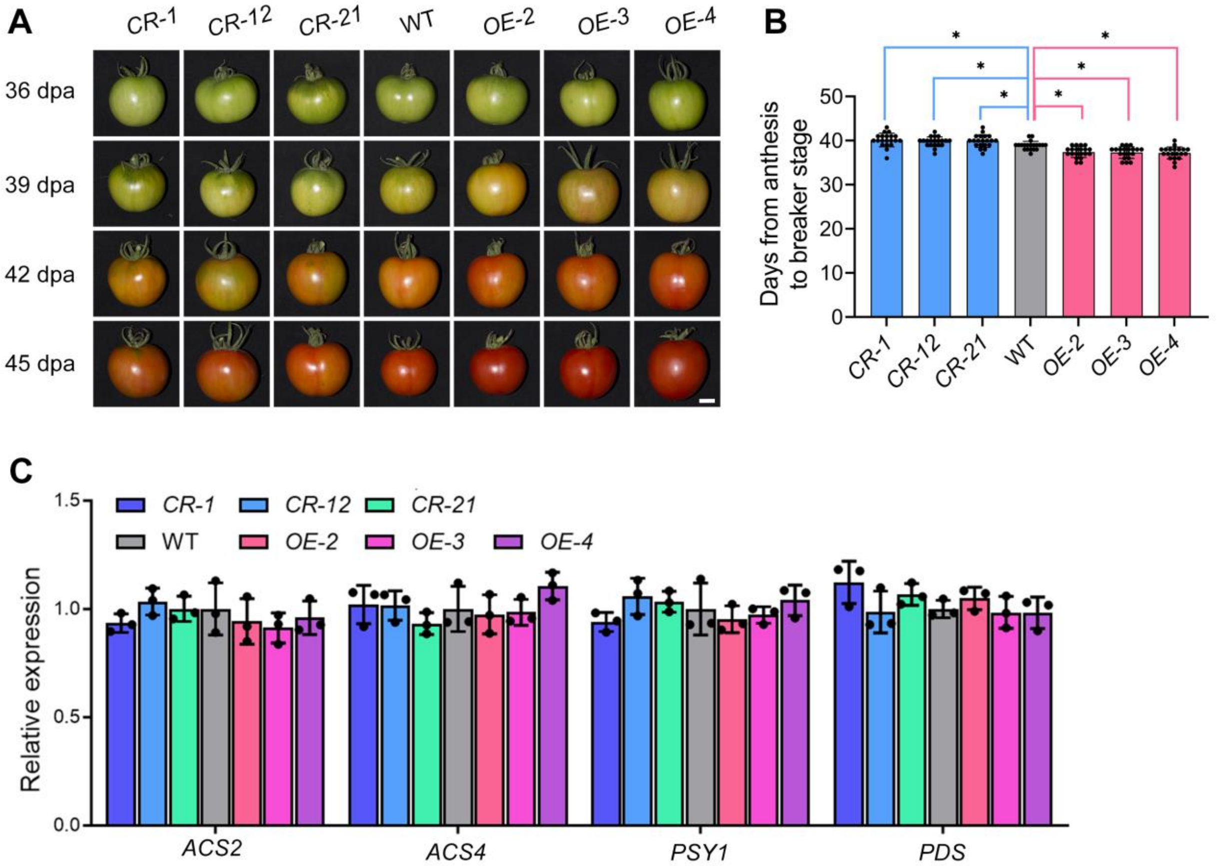

Supplementary Figure S5. Ripening phenotype of *Slcop1-1* mutants and *SlCOP1-1* OE fruits. (A) Representative photograph of fruit from the wild type (WT), *Slcop1-1* mutants, and *SlCOP1-1* overexpression (OE) lines at 36, 39, 42, and 45 days post-anthesis (dpa), corresponding to the mature green (MG), breaker (Br), orange (Or), and red ripe (RR) stages of WT fruits. Scale bar represents 1 cm and applies to all fruit images. (B) Days from anthesis to breaker stage for fruits from WT, *Slcop1-1* mutants, and *SlCOP1-1* OE lines. Data are presented as means ± standard deviation (SD) of 20 fruits. (C) Transcript levels of *ACC synthase 2* (*ACS2*), *ACC synthase 4* (ACS4), *phytoene synthase* (*PSY*), and *phytoene desaturase* (*PDS*) at 39 dpa in WT, *Slcop1-1* mutants, and *SlCOP1-1* OE lines. *Actin* was used as an internal control. The expression level of each gene in WT was defined as 1. Values represent means ± SD of three independent experiments. In (B) and (C), asterisks indicate statistically significant differences (*, $P < 0.05$, Student's *t*-test). *CR-1*, *CR-12*, and *CR-21* represent three independent *Slcop1-1* mutants. *OE-2*, *OE-3*, and *OE-4* represent three independent *SlCOP1-1* OE lines.

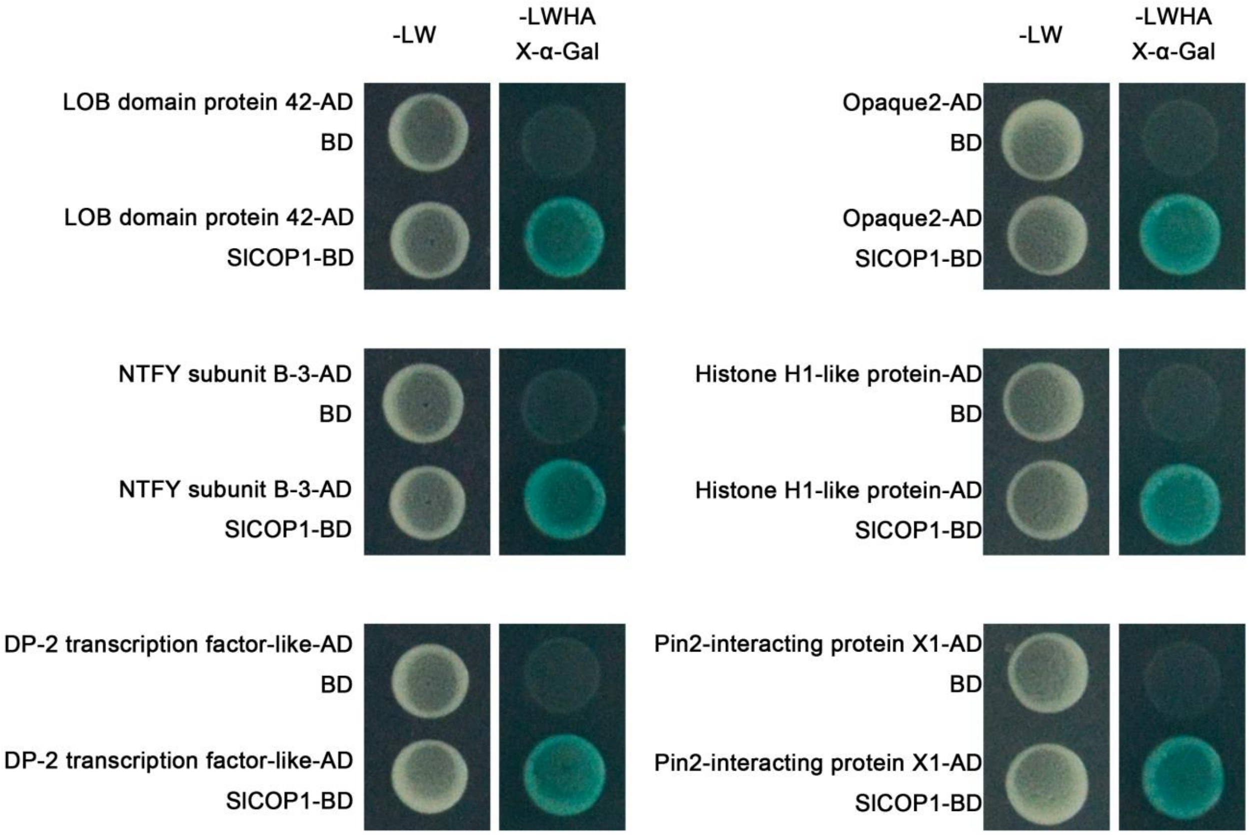

Supplementary Figure S6. Y2H validation confirms the interaction between SlCOP1-1 and candidates identified by Y2H screen. The full-length Open Reading Frames (ORFs) of the candidates were inserted into the pGADT7 (AD) vector, followed by co-transformation with recombinant pGBKT7 (BD)-SlCOP1-1 into yeast. Selection on SD/-Leu/-Trp (-LW), and SD/-Leu/-Trp/-His/-Ade (-LWHA) culture medium with X-α-gal was conducted, while BD/AD-candidate served as a negative control.

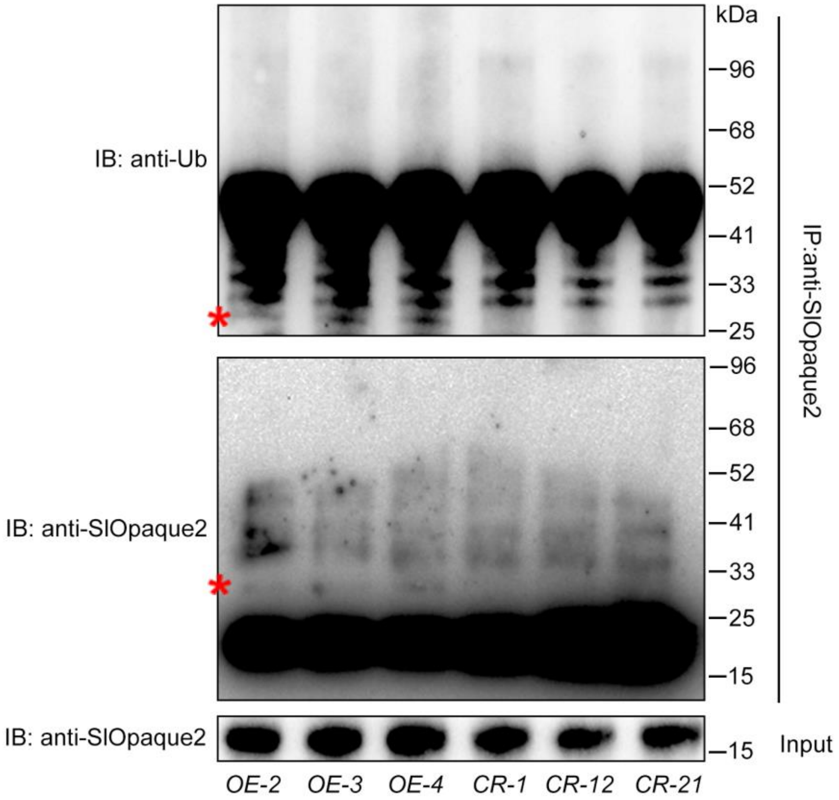

Supplementary Figure S7. SlCOP1-1-mediated ubiquitination of SlOpaque2 in tomato. Total proteins extracted from fruits of *SlCOP1-1* overexpression lines (*OE-2*, *OE-3*, *OE-4*) and *Slcop1-1* mutants (*CR-1*, *CR-12*, *CR-21*) were immunoprecipitated with anti-SlOpaque2 antibody, followed by immunoblotting analysis using anti-Ub or anti-SlOpaque2 antibodies. For the anti-Ub immunoblotting analysis, to avoid interference from the IgG light chain subunit of the anti-SlOpaque2 antibody and the SlOpaque2 main bands (which are similar in size to the SlOpaque2 monoubqituination band), the membrane blotted with proteins below 25 kDa was cut before chemiluminescence detection. IB, immunoblot. IP, immunoprecipitation. Ub, ubiquitin. kDa, kilodalton. The red asterisks indicate the monoubiquitinated bands.

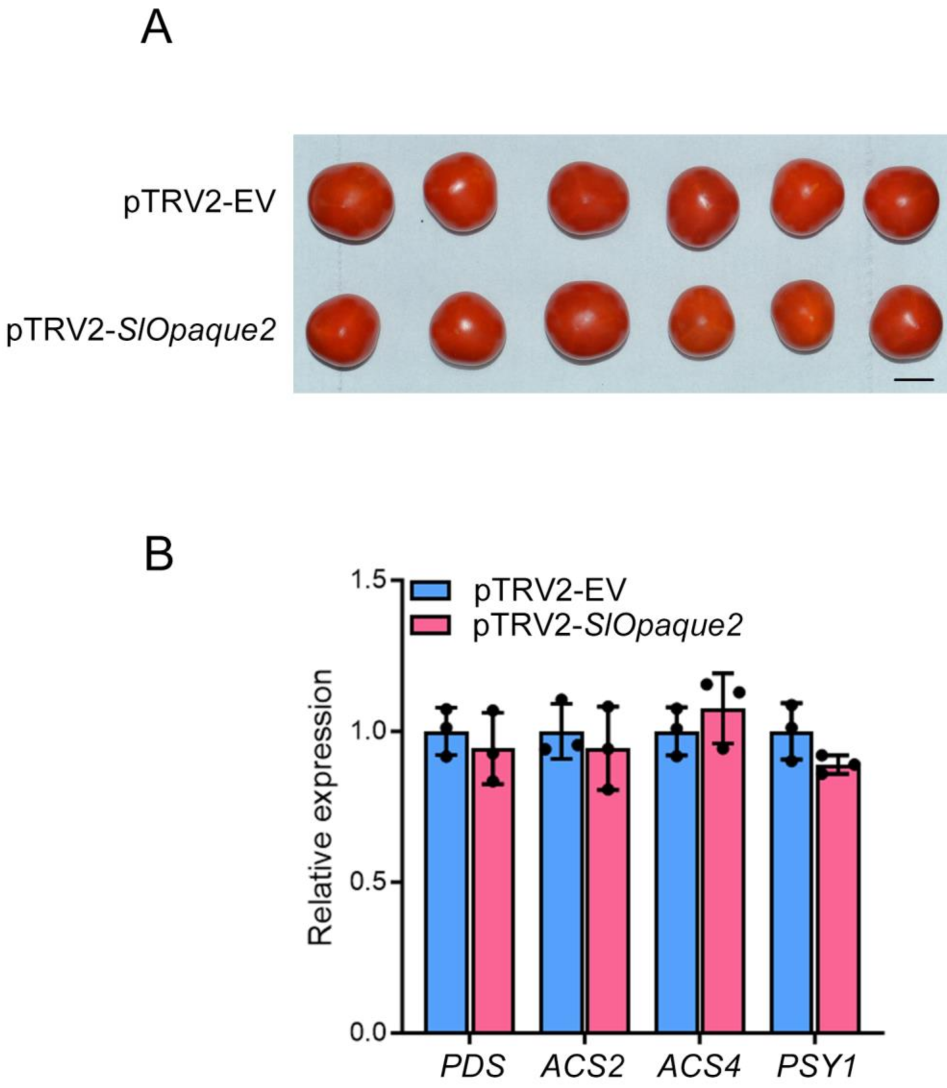

Supplementary Figure S8. Fruit phenotype and ripening-related gene expression analysis in *SlOpaque2*-silenced fruits. Twenty 4-week-old Micro-Tom tomato plants were subjected to needle-injection into the peduncles by *A. tumefaciens* containing pTRV2-SlOpaque2 or pTRV1. (A) Representative photograph of fruit from the control (pTRV2-EV) and pTRV2-*SlOpaque2* plants. Scale bars, 1cm. (B) Transcript levels of *phytoene desaturase* (*PDS*), *ACC synthase 2* (*ACS2*), *ACC synthase 4* (*ACS4*) and *phytoene synthase* (*PSY*). *Actin* was used as an internal control. The expression level of each gene in WT was defined as 1. Values represent means ± standard deviation (SD) of three independent experiments.

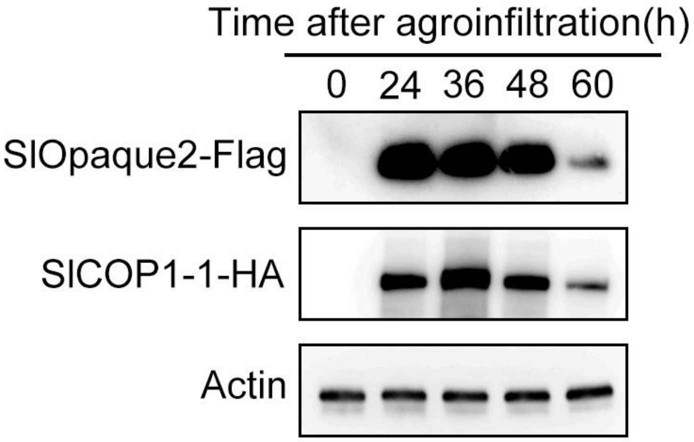

Supplementary Figure S9. Transient co-expression of SlOPaque2 and SlCOP1-1 in *Nicotiana benthamiana* leaves. SlOPaque2 and SlCOP1-1 were transiently co-expressed in *Nicotiana benthamiana* leaves by agroinfiltration for the indicated hours. Total protein extracted from the transformed leaves were subjected to immunoblotting analysis using anti-HA or anti-Flag antibodies. Actin served as the loading control.

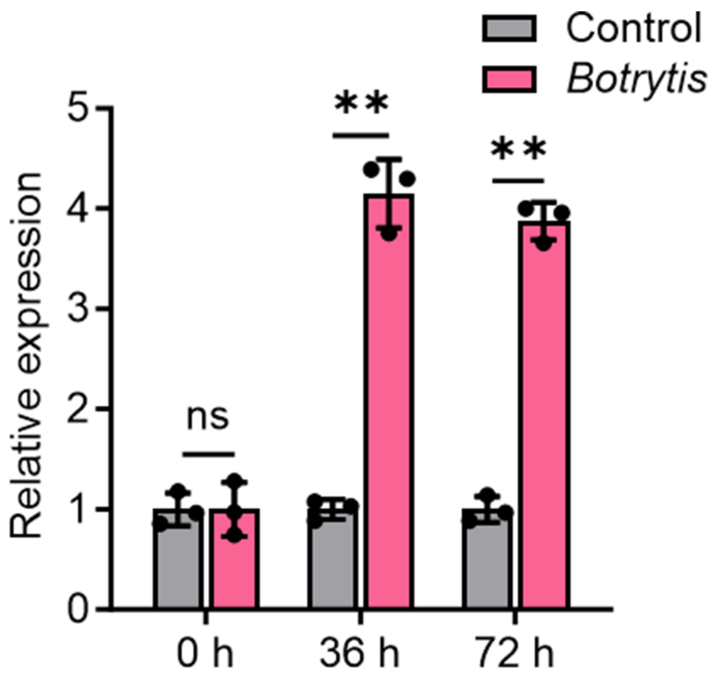

Supplementary Figure S10. *SlCOP1-1* expression in fruits with or without *B. cinerea* inoculation. Fruits were pre-wounded and then inoculated five microliters of conidia suspension (1×10^5 conidia per milliliter). After inoculation, fruit were kept in a growth room at 22°C, with 60%-80% relative humidity and a 16/8 h light/dark photoperiod. Total RNA from fruits were subjected to RT-qPCR analysis. Values represent means ± standard deviation (SD) from three independent experiments. *Actin* served as an internal control. Asterisks indicate statistically significant differences (*, $P < 0.05$; **, $P < 0.01$; Student's *t*-test). ns, not significant. h, hours.

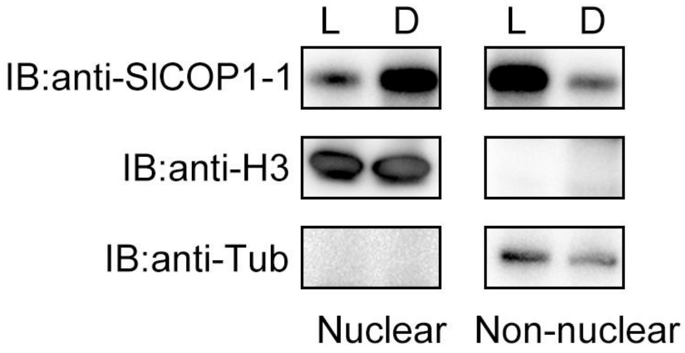

Supplementary Figure S11. Subcellular localization of SlCOP1-1 in tomato fruit cells under light and dark conditions. Fruits were treated with light and dark condition for 12 hours, followed by fruit nuclear isolation and protein extraction as previously described by Wang et al. (2021). Proteins from nuclear and non-nuclear components were analyzed by immunoblotting with an anti-SlCOP1-1 antibody. An anti-histone H3 (anti-H3) and an anti-tubulin (anti-Tub) antibodies were used to indicate different purification fraction. L, light condition; D, dark condition.
